# Predictive value of radiomic features extracted from primary lung adenocarcinoma in forecasting thoracic lymph node metastasis: a systematic review and meta-analysis

**DOI:** 10.1186/s12890-024-03020-x

**Published:** 2024-05-18

**Authors:** Ting Wu, Chen Gao, Xinjing Lou, Jun Wu, Maosheng Xu, Linyu Wu

**Affiliations:** 1 Department of Radiology, The First Affiliated Hospital of Zhejiang Chinese Medical University (Zhejiang Provincial Hospital of Chinese Medicine), 54 Youdian Road, Hangzhou, China; 2 The First School of Clinical Medicine of Zhejiang Chinese Medical University, 548 Binwen Road, Hangzhou, China

**Keywords:** Adenocarcinoma of Lung, Lymphatic Metastasis, Machine learning, Positron Emission Tomography Computed Tomography, Tomography, X-Ray Computed

## Abstract

**Background:**

The application of radiomics in thoracic lymph node metastasis (LNM) of lung adenocarcinoma is increasing, but diagnostic performance of radiomics from primary tumor to predict LNM has not been systematically reviewed. Therefore, this study sought to provide a general overview regarding the methodological quality and diagnostic performance of using radiomic approaches to predict the likelihood of LNM in lung adenocarcinoma.

**Methods:**

Studies were gathered from literature databases such as PubMed, Embase, the Web of Science Core Collection, and the Cochrane library. The Radiomic Quality Score (RQS) and the Quality Assessment of Diagnostic Accuracy Studies-2 (QUADAS-2) were both used to assess the quality of each study. The pooled sensitivity, specificity, and area under the curve (AUC) of the best radiomics models in the training and validation cohorts were calculated. Subgroup and meta-regression analyses were also conducted.

**Results:**

Seventeen studies with 159 to 1202 patients each were enrolled between the years of 2018 to 2022, of which ten studies had sufficient data for the quantitative evaluation. The percentage of RQS was between 11.1% and 44.4% and most of the studies were considered to have a low risk of bias and few applicability concerns in QUADAS-2. Pyradiomics and logistic regression analysis were the most commonly used software and methods for radiomics feature extraction and selection, respectively. In addition, the best prediction models in seventeen studies were mainly based on radiomics features combined with non-radiomics features (semantic features and/or clinical features). The pooled sensitivity, specificity, and AUC of the training cohorts were 0.84 (95% confidence interval (CI) [0.73–0.91]), 0.88 (95% CI [0.81–0.93]), and 0.93(95% CI [0.90–0.95]), respectively. For the validation cohorts, the pooled sensitivity, specificity, and AUC were 0.89 (95% CI [0.82–0.94]), 0.86 (95% CI [0.74–0.93]) and 0.94 (95% CI [0.91–0.96]), respectively.

**Conclusions:**

Radiomic features based on the primary tumor have the potential to predict preoperative LNM of lung adenocarcinoma. However, radiomics workflow needs to be standardized to better promote the applicability of radiomics.

**Trial registration:**

CRD42022375712.

**Supplementary Information:**

The online version contains supplementary material available at 10.1186/s12890-024-03020-x.

## Introduction

Lung cancer is currently the second most common cancer in incidence and the leading cause of cancer-related mortality in the world [1]. Adenocarcinoma is the most common histological subtype [2] and lymph node metastasis (LNM) is the main mode of cancer metastasis. Accurate preoperative prediction of LNM is of great significance in the treatment and prognosis prediction of adenocarcinoma [3]. Currently, diagnostic methods are classified as either invasive or non-invasive. Invasive procedures such as mediastinoscopic biopsy, ultrasound-guided bronchial needle aspiration or lymph node sampling, which will carry risks of postoperative complications to the patient [4, 5]. Non-invasive measures on the other hand are commonly the next best test of choice. Radiological studies like computed tomography (CT), magnetic resonance imaging (MRI) and positron emission tomography/computed tomography (PET/CT), have all demonstrated potential diagnostic efficacy in identifying LNM [6, 7]. Yet, false negative and false positive judgments may be occurred on CT and PET/CT due to some clinical and radiological factors, such as micrometastasis or inflammatory hyperplasia [8, 9]. While MRI is non-radiation and can offers apparent diffusion coefficient characteristics, motion artifacts would limit its assessment in tumor heterogeneity [7, 10].

To improve the efficacy of diagnosis, many studies have relied on radiomics to predict LNM of non-small cell lung cancer [11–13]. Radiomics is a non-invasive technique which can be applied to traditional imaging modalities to extract and quantify radiomic features [14]. Recently, radiomics has already been applied for the identification of malignancy [15] and histological subtypes [16], prediction of gene expression [17], and assessment of treatment response in lung cancer [18]. Radiomic features can be extracted from different regions of interest (ROIs) such as the intratumoral and/or peritumoral areas [19–22]. For example, Das SK et al. improved the performance of predicting cT1N0M0 lung adenocarcinoma by combining features of the intratumor region, the peritumoral region and lymph node [23].

With radiomic approaches becoming more common in medical research, it was hypothesized that radiomic features of primary tumor would be instrumental in predicting the possibility of LNM in lung adenocarcinoma. Therefore, the purpose of this review was to provide a general overview of the methodological quality and evaluate diagnostic performance in radiomics for the prediction of LNM in lung adenocarcinoma.

## Methods

This systematic review and meta-analysis was reported in accordance with the Preferred Reporting Items for Systematic Reviews and Meta-Analyses for Diagnostic Test Accuracy (PRISMA-DTA) guidelines (Additional file 1: Table S1) and was registered on PROSPERO database for systematic reviews (CRD42022375712) [24].

### Database search strategy

A comprehensive search of PubMed, Embase, the Web of Science Core Collection and the Cochrane library was conducted until November 16, 2022. Search terms such as “lung adenocarcinoma”, “machine learning”, “radiomics”, and “lymph node metastasis” were included. The detailed search strategy was described in Table S2 (Additional file 1). No language or publication date restrictions were placed on the initial database search.

### Study selection

Studies were selected if they met all inclusion criteria: (1) patients with lung adenocarcinoma confirmed by pathology; (2) articles based on CT/MRI/PET-CT radiomics to evaluate the likelihood of preoperative LNM; (3) the ROI for segmentation contained the primary tumor; (4) articles were published in English. Studies were excluded if they met any of the following exclusion criteria: (1) case studies, editorials, letters, review articles and conference abstracts; (2) studies not in the field of interest.

### Data extraction

Two independent investigators firstly extracted the following information from each selected study: (1) study details: first author, publication year, country of origin, study design; (2) patient details: the source of data acquisition, criteria for lymph node staging, diameter and density of primary tumor, diagnostic method of LNM, number of patients and negative/positive LNM in the training/internal validation/external validation cohort, clinical stage; (3) imaging details: imaging modality; (4) radiomic details: segmentation method and software, ROI, radiomic feature extraction software and method, number of radiomic features extracted, type of radiomic features extracted, type of models constructed, the best performance model, number of radiomic/non-radiomic features included in the best performance model; (5)diagnostic performance: sensitivity, specificity and area under the curve (AUC)/concordance index (C-index) of the prediction models.

If more than one predictive model was included in a study, the radiomics model with the highest AUC/C-index in the training and validation cohort was included in the quantitative evaluation, respectively [25, 26]. If an internal validation cohort and an external validation cohort were included in a study, we included data from both cohorts.

### Risk of bias assessment

The Radiomic Quality Score (RQS) [27] was used to evaluate the procedural validity of each study (Additional file 1: Table S3). The RQS provided rigorous evaluation criteria and reporting guidelines for radiomic studies [27]. The total score ranged from -8 to 36, and sixteen items are assigned corresponding scores [27]. The Quality Assessment of Diagnostic Accuracy Studies (QUADAS-2) [28] was used to determine the risk of bias and the applicability of each included study (Additional file 1: Table S4). The QUADAS-2 tools was first divided into two broad categories: the risk of bias and the applicability concerns [28]. The former included features such as patient selection, index test, reference standard, flow and timing [28]. The latter examined similar parameters with patient selection, index test and reference standard [28]. Based on basic answers of "yes", "no", or "unclear" for each item, the level was rated as "low", "high", or "unclear" [28]. The RQS and QUADAS-2 were used to evaluate the quality of the literature independently by two authors. Discrepancies were rediscussed and evaluated to reach a consensus.

### Statistical analysis

Firstly, we extracted sample size, sensitivity, and specificity of the best radiomics models in the training and validation cohorts from the studies. Then the number of true positives, false positives, false negatives, and true negatives were calculated by Review Manager 5.4.

Quantitative evaluation was performed using the midas command in Stata 17.0 software. Pooled sensitivity, specificity, positive likelihood ratio (PLR), negative likelihood ratio (NLR), diagnostic odds ratio (DOR), and AUC were calculated, and summary receiver operating characteristic curve (SROC) was created. Heterogeneity was assessed using Cochrane Q-test (two-sides *p* < 0.05 was considered statistically significant) and I^2^ statistic (I^2^ values of 25%, 50% and 75% represent low, moderate and high heterogeneity, respectively) from forest plots [29]. Spearman rank coefficients was performed to determine whether there was heterogeneity caused by threshold effect. The sources of heterogeneity were further analyzed by subgroup and univariate meta-regression analyses.

## Results

### Literature search and extraction

**Fig. 1:**
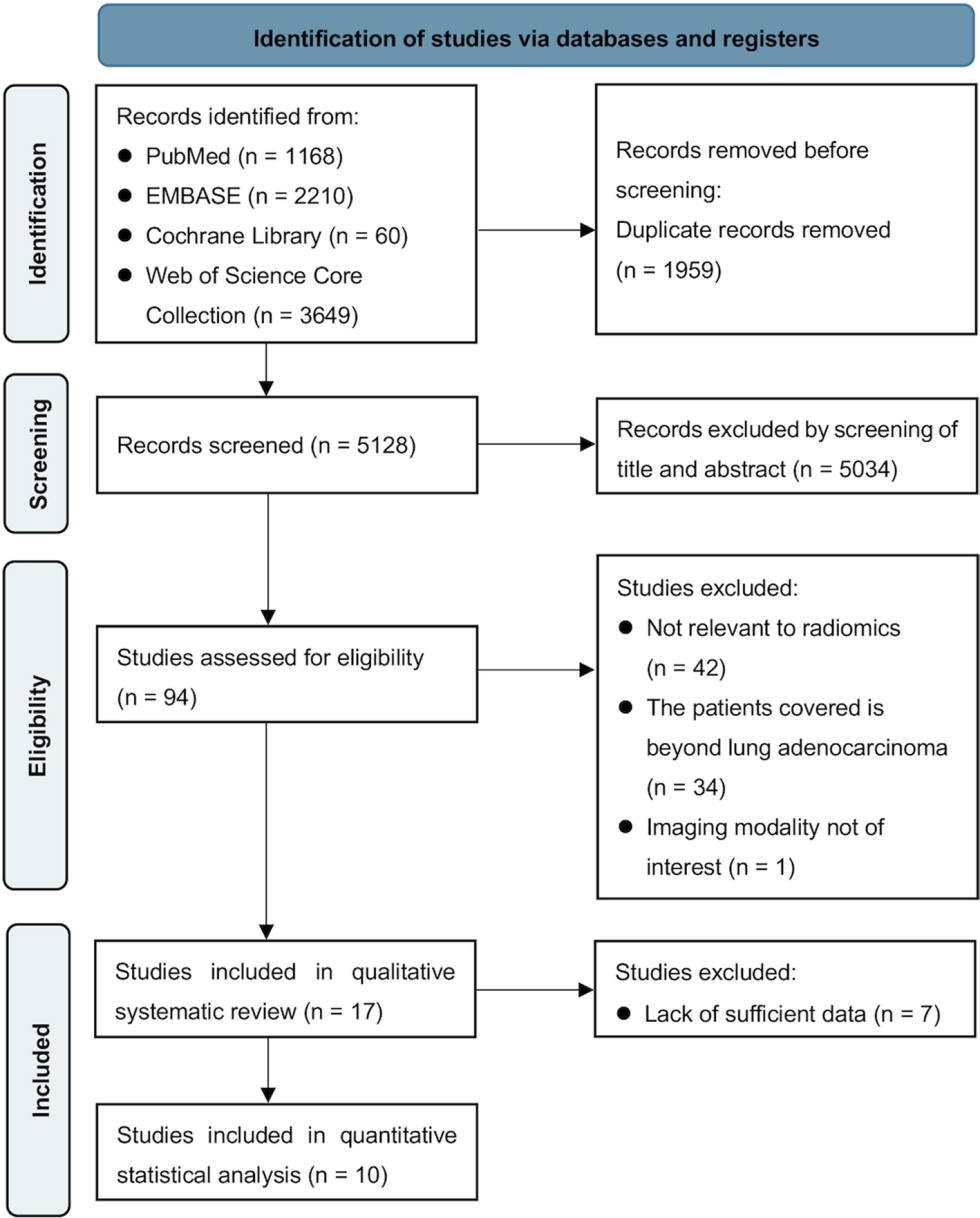
Flowchart of the study screening and selection process

A total of 7087 studies were obtained by the search strategy of which 1959 remained after removing duplicates. After, 5034 articles did not meet the inclusion criteria based on title and abstract and 94 studies were examined in full text. Among them, 42 studies were not related to radiomics, 34 studies covered patients beyond lung adenocarcinoma, and the imaging modality of 1 study was not of interest (ultrasound). Finally, this systematic review involved 17 studies containing a total of 7,117 patients [23, 30–45]. Seven studies [30, 31, 35, 37–39, 44] were excluded due to lack of sufficient data, and 10 studies [23, 32–34, 36, 40–43, 45] were included in the meta-analysis. Figure 1 illustrates the PRISMA flow chart for the included studies in this review.

### Patient and study characteristics

Table 1 presents the basic characteristics for all 17 retrospective studies which were published between 2018 and 2022 [23, 30–45]. Most of the studies (14/17, 82.4%) were derived from one center [30–39, 41, 42, 44, 45]. And almost all of the studies (16/17, 94.1%) were from China [23, 30, 32–45], except for one from the United States [31]. The included studies (11/17, 64.7%) [23, 31–34, 36, 37, 39–41, 44] usually used the 8th edition of tumor-node-metastasis staging system as the standard for lymph node staging [46].

**Table 1:** Characteristics of included studies

Study ID	Country^f^	Study Design	Criteria for LN staging	Diameter and density of primary tumor	Clinical stage	Imaging Modality	Method of LNM diagnosed	No. of patients	Training		Internal validation		External validation	
									LN +	LN-	LN +	LN-	LN +	LN-
2018 Gu [30]	China	R, single-center	7th	≤ 20 mm; /	cT1aN0M0	CT^c^	surgical resection	811	41	460	/	/	38^e^	272^e^
2018 Liu [31]	USA	R, single-center	8th	/; GGO, PSN, SN	cN0	CT^b^	surgical resection	187	34	153	/	/	/	/
2018 Yang [32]	China	R, single-center	8th	/; SN	/	CT^c,d^	surgical resection	159	49	57	25	28	/	/
2018 Zhong [33]	China	R, single-center	8th	≤ 70 mm; /	cT1–3N0M0	CT^c^	surgical resection	492	78	414^a^	/	/	/	/
2019 Wang [34]	China	R, single-center	8th	≤ 30 mm; GGO, PSN, SN	cT1	CT^b^	surgical resection	366	49	193	22	102	/	/
2019 Yang [35]	China	R, single-center	/	< 40 mm; /	cT1–2aN0	CT^b^	surgical resection	1202	141	739	43	279^a^	/	/
2020 Zhu [36]	China	R, single-center	8th	≤ 30 mm; PSN, SN	cT1N0M0	CT^b^, PET/CT	surgical resection	256	39	217	/	/	/	/
2021 Das [23]	China	R, two-center	8th	≤ 30 mm; GGO, PSN, SN	cT1N0M0	CT^d^	surgical resection	216	39	74	17	33	31	22
2021 Li [37]	China	R, single-center	8th	/; PSN, SN	/	CT^c^	surgical resection	556^a^	228	162	91	75	/	/
2021 Ran [38]	China	R, single-center	/	/; /	/	CT^b^	surgical resection	300	100	100	20	20	30^e^	30^e^
2021 Wang [39]	China	R, single-center	8th	/; SN	cN0	PET/CT	surgical resection	370	66	170	/	/	32^e^	102^e^
2021 Zhang [40]	China	R, two-center	8th	≤ 40 mm; SN	cT1-2N0M0	CT^d^	surgical resection	244	34	126	/	/	21	63
2022 Chang [41]	China	R, single-center	8th	≤ 30 mm; PSN, SN	cT1	PET/CT	surgical resection	528	105	266	44	113	/	/
2022 Chen [42]	China	R, single-center	/	/; GGO, PSN, SN	/	CT^c^	surgical resection	207	35	88	15	69	/	/
2022 Dai [43]	China	R, two-center	/	≥ 10 mm; PSN, SN	cTxNxMx	PET/CT	surgical resection and LN sampling	320	63	138	27	60	18	14
2022 Lv [44]	China	R, single-center	8th	≤ 30 mm; /	cT1M0	PET/CT	surgical resection and CT follow-up validation	183	26	104	9	44	/	/
2022 Ma [45]	China	R, single-center	/	/; /	/	CT^d^	surgical resection	720^a^	133	356	33	90	33^e^	75^e^

*CT* computed tomography, *GGO* ground glass opacity, *HU* hounsfield unit, *LN* lymph node, *LN* + positive lymph node metastasis, *LN-* negative lymph node metastasis, *PSN* part solid nodule, *SN* solid nodule, *R* retrospective, *USA* The United States of America,* 7th* the seventh edition of tumor-node-metastasis (TNM), *8th* the eighth edition of TNM, *PET/CT* fluorine-18 fluorodeoxyglucose positron emission tomography/computed tomography

^a^There are inconsistencies in the data in the original literature

^b^With either contrast enhanced CT or unenhanced CT

^c^With unenhanced CT

^d^With contrast enhanced CT

^e^Patients in the validation cohort were recruited from the same center

^f^Based on corresponding authors' affiliations

All studies relied on surgical resection for the diagnosis of LNM. One study also included lymph node sampling [43], and one study included CT follow-up validation [44]. The number of patients included ranged from 159 to 1202. Eleven studies (11/17, 64.7%) [23, 32, 34, 35, 37, 38, 41–45] had internal validation cohorts and eight studies [23, 30, 38–40, 43–45] had external validation cohorts. Eight studies selected patients with clinical stage N0 at enrollment [23, 30, 31, 33, 35, 36, 39, 40].

### Radiomics workflow

CT was the primary imaging modality in 13 studies [23, 30–38, 40, 42, 45]. In addition, ^18^F-PET/CT was used in five studies [36, 39, 41, 43, 44]. The ROIs were manually segmented in 11 studies [23, 30, 33, 34, 36–38, 40, 42, 44, 45], semi-automatically in five studies [31, 35, 39, 41, 43] and fully automatically in one study [32] (Table 2). There were eight types of ROI segmentation software, among which the most frequently used was ITK-SNAP [23, 37, 41, 44, 45]. All studies included primary tumors in their ROI segmentation [23, 30–45].

**Table 2:** Radiomics workflow for the included studies

Study ID	Segmentation Method and software	Regions of interest	No. Radiomic features extracted and extraction software	The type of radiomics features extracted	Radiomics features selection methods	Type of prediction models constructed	No. radiomics features in best model	No. non-radiomics features in best model
2018 Gu [30]	Manual; in-house software (Medical Imaging Solution for Segmentation and Texture Analysis)	primary tumor	NA; NA	first order, shape, texture (GLCM)	Multivariate logistic regression, 5-fold cross validation	model_Nomogram^e^	3	1
2018 Liu [31]	Semi-automated; Definiens Developer XD	primary tumor	219; Definiens Developer XD	first order, shape, texture (GLRLM, laws), high order(wavelet)	Pearson correlation analysis, FDR, PCA, Univariate and multivariate logistic regression, Backward elimination method	model_Semantic; model_Radiomic; model_Combined^e^	1	1
2018 Yang [32]	Autometed; 3D U-Net model	primary tumor	94; Pyradiomics	first order, shape, texture (GLCM, GLRLM, GLSZM)	LASSO, Multivariate logistic regression	model_Radiomic; model_Nomogram^e^	14	1
2018 Zhong [33]	Manual; in-house software (Analysis Kit)	primary tumor	300; Mazda	first order, texture (GLCM, GLRLM), high order(wavelet)	ICC, Relief analysis, Single linkage (nearest neighbor) hierarchic clustering analysis, PCA	model_Clinical+Semantic; model_Radiomic^e^	4	0
2019 Wang [34]	Manual; Medical Imaging Interaction Toolkit (MITK)	primary tumor and peritumoral volume^a^	3892; Pyradiomics	first order, shape, texture (GLCM, GLSZM, GLRLM, NGTDM, GLDM), high order(wavelet)	mRMR, LASSO, ICC, Pearson correlation analysis	model_Semantic; model_Radiomic(GTV, PTV, GPTV); model_Nomogram^e^	5	6
2019 Yang [35]	Semi-automated; 3D slicer	primary tumor	1078; Pyradiomics	first order, shape, texture (GLCM, GLRLM, GLSZM)	mRMR, LASSO, Unsupervised cluster analysis, PCA	model_Radiomic^e^	5	2
2020 Zhu [36]	Manual; NA	primary tumor	NA; MaZda	first order, texture (GLRLM, GLCM)	Univariate and multivariate logistic regression	model_Radiomic (Mean, Skewness, Entropy); model_Semantic (TV, AVG^e^)	0	1
2021 Das [23]	Manual; ITK‐SNAP	primary tumor, peritumoral volume^b^ and LN	1584; Artificial Intelligence Kit software (A.K.)	first order, shape, texture(GLCM, GLSZM, GLRLM)	ICC, Spearman correlation analysis, LASSO logistic regression	model_Clinical; model_ Radiomic (GTV, PTV, GPTV, LN, GPTV + LN); model_Nomogram^e^	6	2
2021 Li [37]	Manual; ITK‐SNAP	primary tumor and adjacent pleura^c^	1300; Pyradiomics	first order, shape, texture(GLCM)	ICC, Pearson correlation analysis, Minimum Akaike’s information criterion, Optimal subset, Typical features	model_ Radiomic (Primary tumor, Pleura around the tumor, Combined); model_Combined^e^	32^d^	2
2021 Ran [38]	Manual; NA	primary tumor	1288; Pyradiomics	first order, shape, texture (GLCM, GLSZM, GLRLM, NGTDM, GLDM), high order(wavelet)	T-test, Extra-trees	model_Radiomic; model_Deep learning; model_Semantic; model_Nomogram^e^	8	2
2021 Wang [39]	Semi-automated; Region Studio	primary tumor	107; Region Studio	first order, shape, texture(GLCM, GLSZM, GLRLM, NGTDM, GLDM)	ICC, LASSO, Multivariate logistic regression	model_Nomogram^e^	4	2
2021 Zhang [40]	Manual; NA	primary tumor	851; Pyradiomics	first order, shape, texture(GLCM, GLRLM, GLSZM, NGTDM), high order(wavelet)	ICC, LASSO, Multivariate logistic regression	model_Semantic; model_Radiomic; model_Nomogram^e^	3	1
2022 Chang [41]	Semi-automated; ITK‐SNAP	primary tumor	402; Artificial Intelligence Kit software (AK)	first order, shape, texture(GLCM, GLRLM, GLSZM)	mRMR, LASSO, ICC	model_Radiomic(PET, CT, PET/CT); model_Semantic; model_Nomogram^e^	10	2
2022 Chen [42]	Manual; NA	primary tumor	35; NA	first order, texture (GLCM)	Univariate and multivariate logistic regression, LASSO	model_Clinical; model_Semantic; model_Radiomic; model_Combined^e^	2	3
2022 Dai [43]	Semi-automated; NA	primary tumor	190; LIFEx software	first order, shape, texture(GLCM, GLRM, NGLDM, GLZLM)	Mann–Whitney U test, LASSO	model_Semantic; model_Radiomic; model_Combined^e^	10	2
2022 Lv [44]	Manual; ITK‐SNAP	primary tumor	974; MATLAB	first order, shape, texture(GLCM, GLRLM, GLSZM, GLDZM, NGTDM, NGLDM)	ICC, Information evaluation, Univariate logistic regression analysis, LASSO, Pearson correlation analysis, 50 times fivefold cross-validation	model_Clinical-Semantic; model_Radiomic; model_Combined; model_Clinical-Semantic + ENN; model_Radiomic + ENN; model_Clinical-Semantic-Radiomic+ ENN^e^	8	4
2022 Ma [45]	Manual; ITK‐SNAP	primary tumor	1210; Pyradiomics	first order, shape, texture(GLCM, GLRLM, GLSZM, GLDM, NGTDM)	ICC, Variance filtering method, Student's t-test or Mann–Whitney test, Multivariable logistic regression	model_Semantic; model_Radiomic; model_Deep Learning; model_Combined(Clinical-Semantic, Clinical-Semantic-Radiomics, Clinical-Semantic-Deep Learning, Clinical-Semantic-Radiomic-Deep Learning^e^)	18	6

*AVG* average CT values of whole tumor, *CT* computed tomography, *FDR* false discovery rate, *GLCM* gray level co-occurrence matrix, *GLDM* gray level dependence matrix, *GLDZM* gray level distance zone matrix, *GLRLM* gray level run length matrix, *GLSZM* gray level size zone matrix, *GPTV* gross and peritumoral volume, *GTV* gross tumor volume, *ICC* intra-class correlation coefficients, *LASSO* least absolute shrinkage and selection operator, *mRMR* minimum-redundancy-maximum-relevance, *NA* not available, *NGLDM* neighborhood grey level dependence matrix, *NGTDM* neighbouring gray tone difference matrix, *PCA* principle component analysis, *PET/CT* fluorine-18 fluorodeoxyglucose positron emission tomography/computed tomography, *PTV* peritumoral volume

^a^15mm around the tumor

^b^5 mm around the tumor

^c^Adjacent pleural regions of interest delineation was defined as two lines tangent to the edges of the tumor, intersecting the visceral pleura at 90°

^d^There are inconsistencies in the data in the original literature

^e^The best performance model of all the prediction models constructed in the study

A total of seven different software was applied for the extraction of radiomic features in each study, among which Pyradiomics was the most used [32, 34, 35, 37, 38, 40, 45] (Table 2). The common methods of radiomic feature selection were logistic regression analysis [23, 30–32, 36, 39, 40, 42, 44, 45] and least absolute shrinkage and selection operator method [23, 32, 34, 35, 39–44]. The number of radiomics features included ranged from 1 to 32 in each of the best models, except for one study in which the best model included only semantic features without radiomic features [36]. The types of prediction models constructed ranged from 1 to 7, and most of the best models (15/17, 88.2%) were models that combined radiomic and non-radiomic features (semantic features and/or clinical features) (Additional file 1: Table S5) [23, 30–32, 34, 35, 37–45].

### Quality assessment

**Fig. 2:**
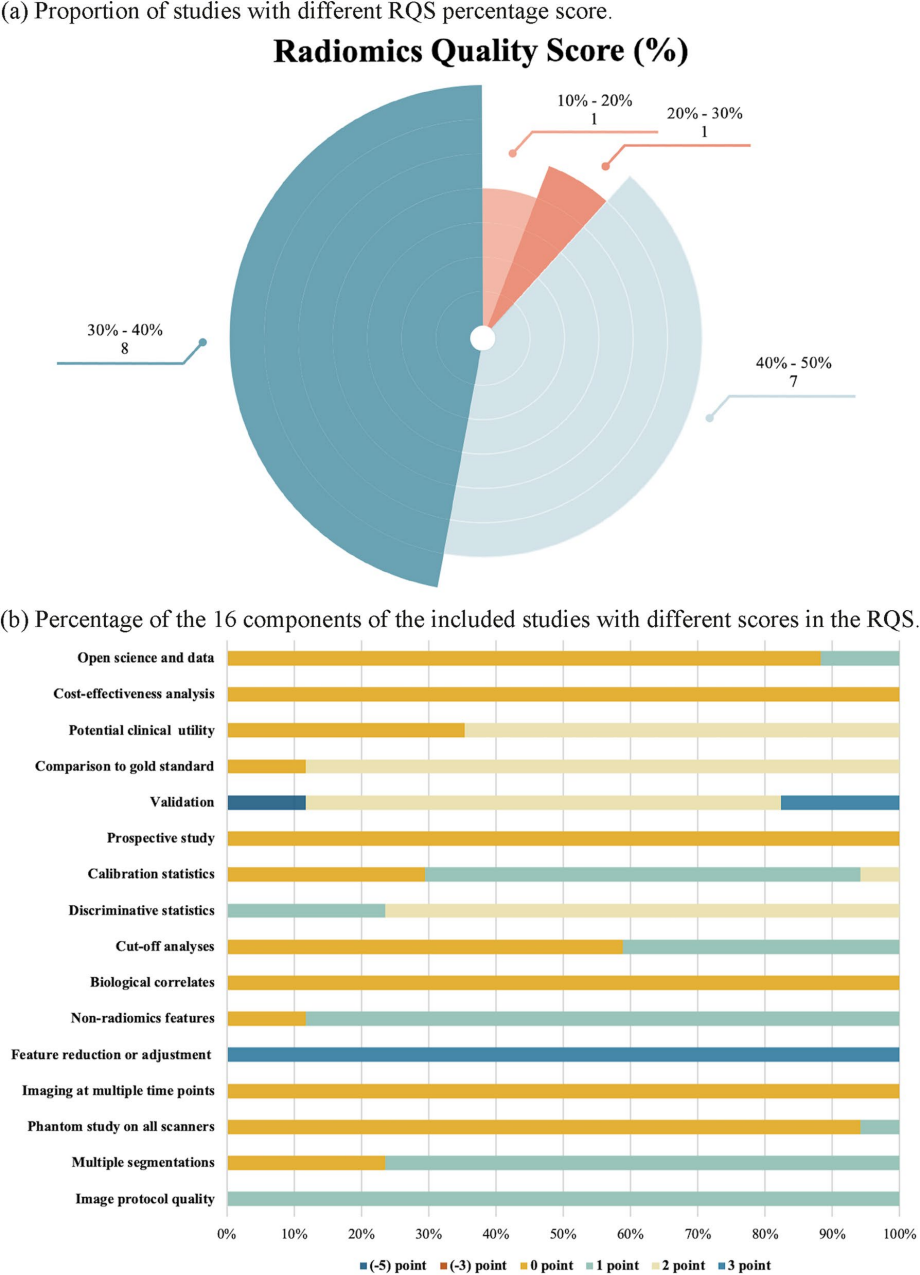
Qualitative quality assessment evaluated through the Radiomics Quality Score (RQS) tool. **a** Proportion of studies with different RQS percentage score. **b** Percentage of the 16 components of the included studies with different scores in the RQS

The overall RQS and percent RQS for each study are presented in Table 3 and Fig. 2, along with the scores for the individual components. The median RQS total scores was 14 (range 4 – 16) and 38.9% (range 11.1% – 44.4%). Most studies (8/17, 47.1%) had RQS scores between 30% and 40% (Fig. 2a). No study scored in the four items of “Cost-effectiveness analysis”, “Prospective study” “Biological correlates” and “Imaging at multiple time points” (Fig. 2b).

**Table 3:** Radiomic quality scores for all included studies

Study ID	Image protocol quality (0—2)	Multiple segmentations (0—1)	Inter-scanner Differences (0—1)	Imaging at multiple time points (0—1)		Feature reduction or adjustment (-3—3)	Non-radiomics features (0—1)	Biological correlates (0—1)	Cut-off analyses (0—1)
2018 Gu [30]	1	1	0	0		3	1	0	1
2018 Liu [31]	1	1	0	0		3	1	0	0
2018 Yang [32]	1	0	0	0		3	1	0	1
2018 Zhong [33]	1	1	0	0		3	0	0	1
2019 Wang [34]	1	1	0	0		3	1	0	1
2019 Yang [35]	1	1	0	0		3	1	0	0
2020 Zhu [36]	1	1	0	0		3	0	0	1
2021 Das [23]	1	0	1	0		3	1	0	0
2021 Li [37]	1	1	0	0		3	1	0	0
2021 Ran [38]	1	0	0	0		3	1	0	0
2021 Wang [39]	1	1	0	0		3	1	0	0
2021 Zhang [40]	1	1	0	0		3	1	0	0
2022 Chang [41]	1	1	0	0		3	1	0	1
2022 Chen [42]	1	1	0	0		3	1	0	1
2022 Dai [43]	1	1	0	0		3	1	0	0
2022 Lv [44]	1	0	0	0		3	1	0	0
2022 Ma [45]	1	1	0	0		3	1	0	0
**Median score**	**1**	**1**	**0**	**0**		**3**	**1**	**0**	**0**
Study ID	Discriminative statistics (0—2)	Calibration statistics (0—2)	Prospective study (0—7)	Validation (-5—5)	Comparison to gold standard (0—2)	Potential clinical utility (0—2)	Cost-effectiveness analysis (0—1)	Open science and data (0—4)	Total points (-12—36)
2018 Gu [30]	2	2	0	2	2	0	0	0	15
2018 Liu [31]	2	1	0	-5	2	2	0	0	8
2018 Yang [32]	2	1	0	2	2	2	0	0	15
2018 Zhong [33]	2	0	0	2	2	0	0	0	12
2019 Wang [34]	2	0	0	2	2	0	0	0	13
2019 Yang [35]	2	1	0	2	2	0	0	0	13
2020 Zhu [36]	1	0	0	-5	2	0	0	0	4
2021 Das [23]	2	1	0	3	2	2	0	0	16
2021 Li [37]	1	1	0	2	0	2	0	0	12
2021 Ran [38]	2	1	0	2	2	2	0	0	14
2021 Wang [39]	1	1	0	2	2	2	0	0	14
2021 Zhang [40]	2	1	0	3	2	2	0	0	16
2022 Chang [41]	2	1	0	2	2	2	0	0	16
2022 Chen [42]	2	0	0	2	0	2	0	0	13
2022 Dai [43]	2	1	0	3	2	2	0	0	16
2022 Lv [44]	2	0	0	2	2	0	0	1	12
2022 Ma [45]	1	1	0	2	2	2	0	1	15
**Median score**	**2**	**1**	**0**	**2**	**2**	**2**	**0**	**0**	**14**

**Fig. 3:**
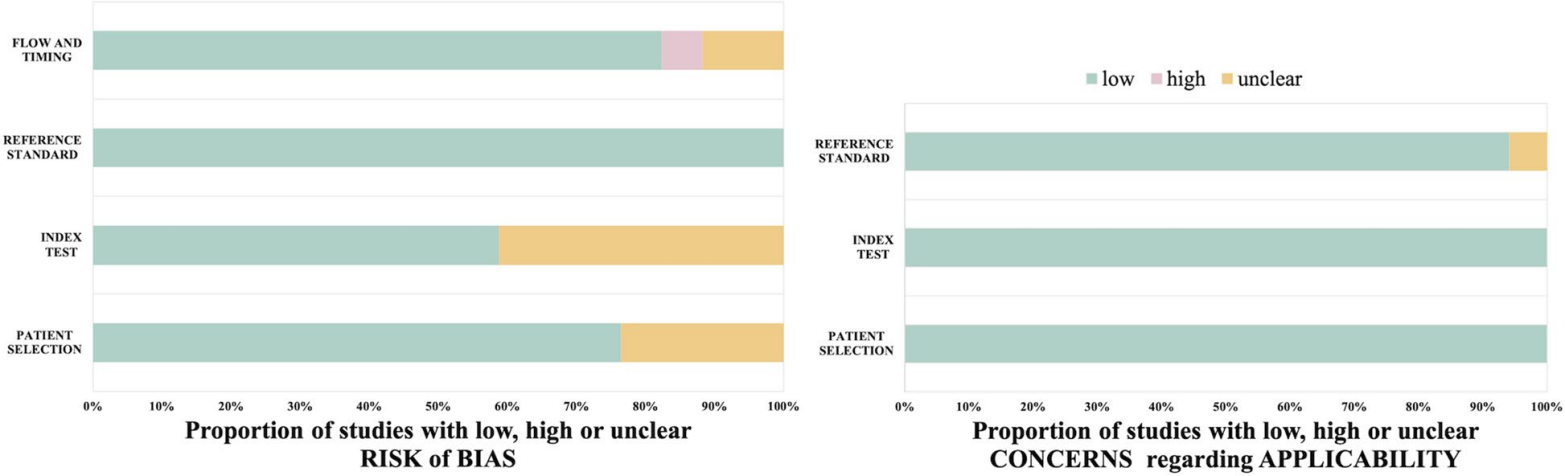
The percentage of the Quality Assessment of Diagnostic Accuracy Studies (QUADAS-2) scoring criteria

The distribution of the QUADAS-2 scores for each included study was shown in Table S6 (Additional file 1) and Fig. 3. The risk of bias in patient selection was low in 13 studies and unclear in 4 studies. The risk of bias for the index test was low in 10 studies and unclear in 7 studies. The risk of bias for the reference standard test was low in 17 studies. The risk of bias for flow and timing was low in 14 studies, unclear in 2 studies, and high in 1 study. Most studies were assessed as having a low risk of bias and minimal concerns regarding applicability.

## Data analysis

### Diagnostic performance

**Fig. 4:**
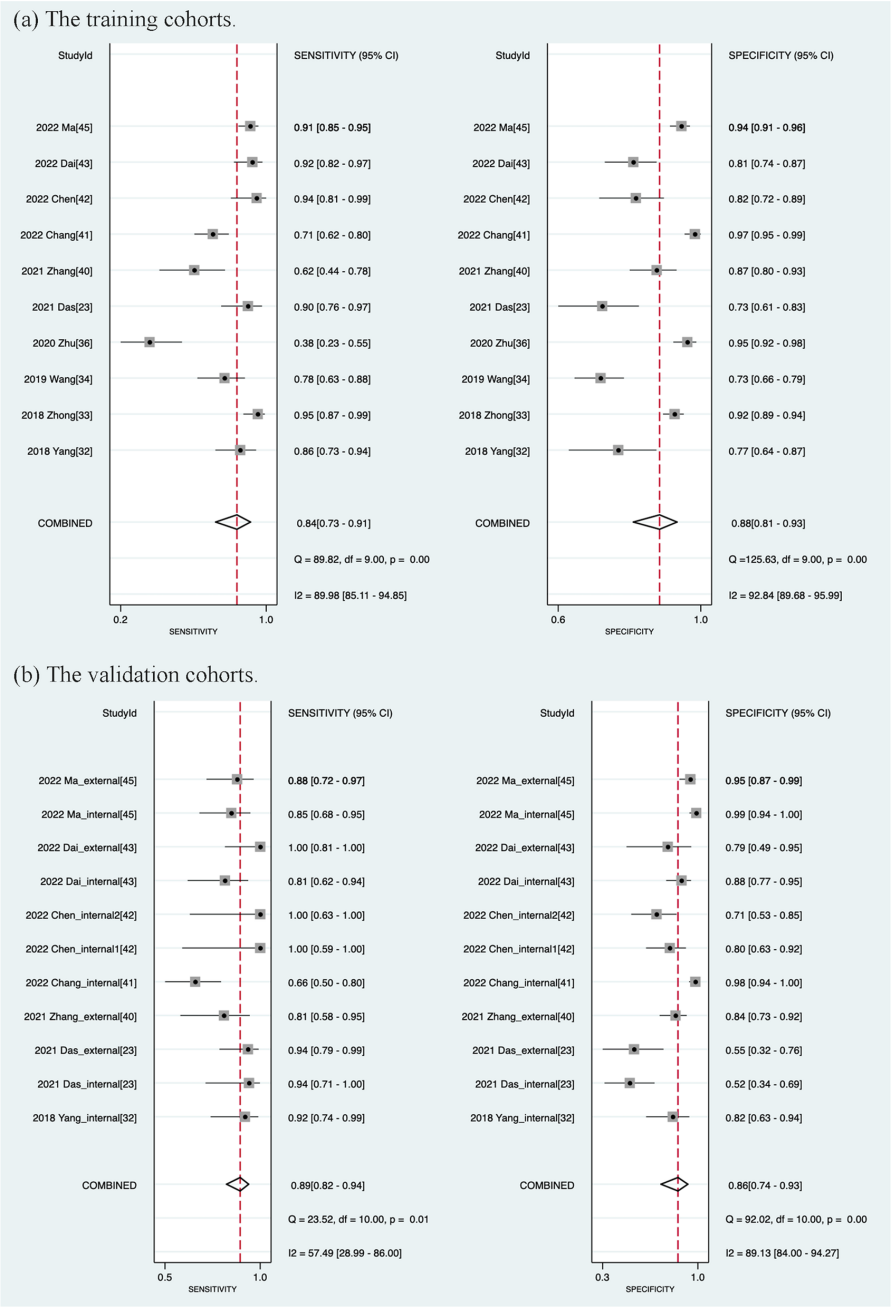
Coupled Forest plots of pooled sensitivity and specificity. **a** The training cohorts. **b** The validation cohorts. (internal: an internal validation cohort; external: an external validation cohort)

**Fig. 5:**
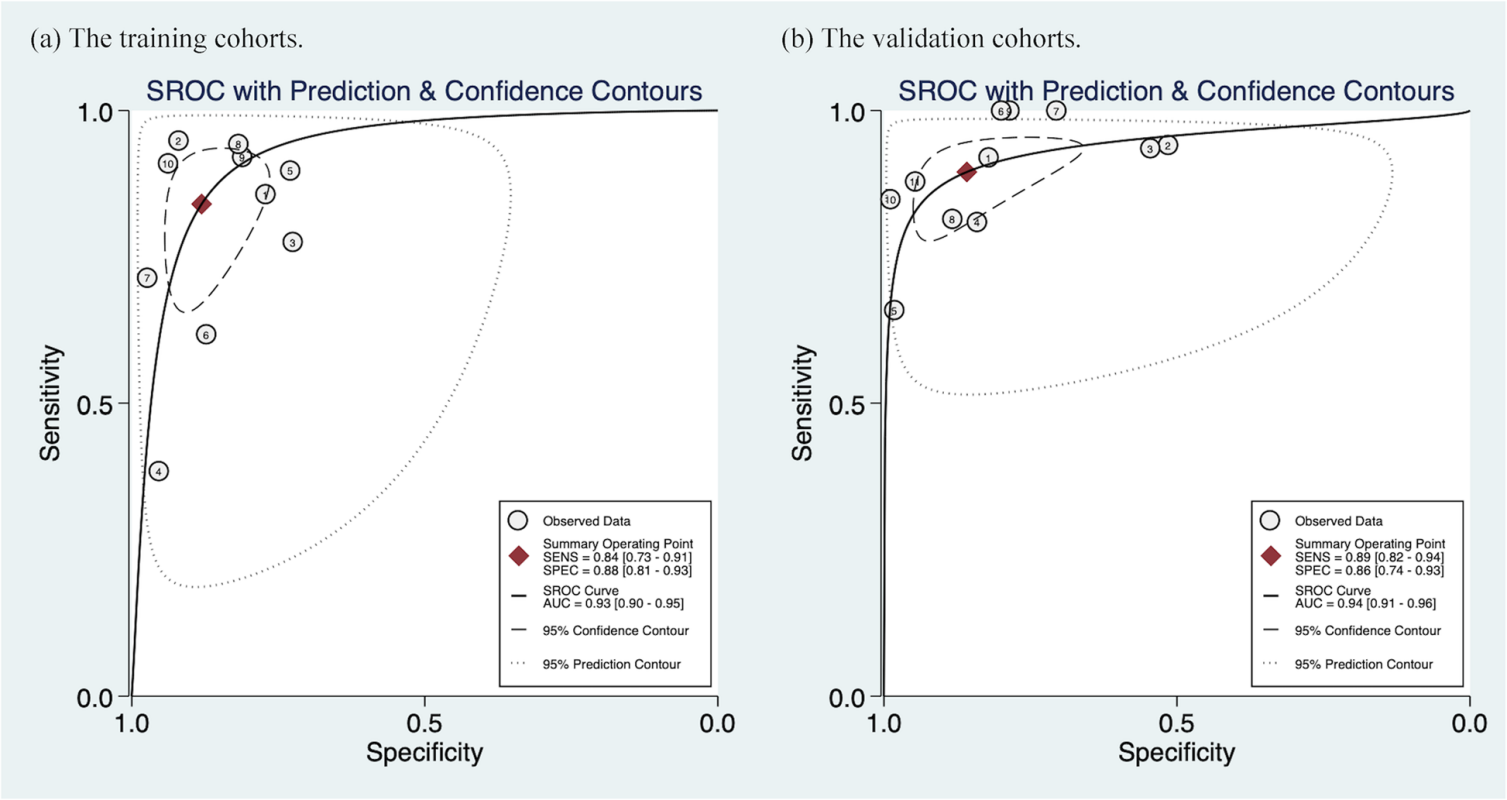
Summary receiver operating characteristic curves (SROC) of the diagnostic performance. **a** The training cohorts. **b** The validation cohorts

The diagnostic efficacy of each study will be presented in Table S7-S9 (Additional file 1:). Ten studies were included in this meta-analysis, in which the pooled sensitivity, specificity, PLR, NLR, DOR and AUC in the training cohorts were 0.84 (95% CI [0.73–0.91]), 0.88 (95% CI [0.81–0.93]), 7.0 (95% CI [4.5–11.0]), 0.18 (95% CI [0.11–0.31]), 39 (95% CI [19–78]), 0.93 (95% CI [0.90–0.95]), respectively. Meanwhile, three studies did not evaluate the diagnostic performance of the validation cohorts due to the lack of validation cohorts [33, 34, 36]. The pooled sensitivity, specificity, PLR, NLR and DOR of 11 internal and external validation cohorts from 7 studies were 0.89 (95%CI [0.82–0.94]), 0.86 (95% CI [0.74–0.93]), 6.3 (95% CI [3.4–11.8]), 0.12 (95% CI [0.08–0.20]), 52 (95% CI [27–97]), 0.94 (95% CI [0.91–0.96]), respectively. Figure 4 and Fig. 5 show the forest plots and SROC plots for the training and validation cohorts, respectively. High heterogeneity was observed in the sensitivity and specificity of the training cohorts (*p* ≤ 0.01, I^2^ = 89.98; *p* ≤ 0.01, I^2^ = 92.84). Since only seven studies involved the validation cohorts, we mainly explored the sources of heterogeneity of the ten studies for the training cohorts. Spearman correlation coefficient was -0.45 (*p* = 0.17), indicating that heterogeneity due to threshold effects may be low.

### Investigation of heterogeneity

**Fig. 6:**
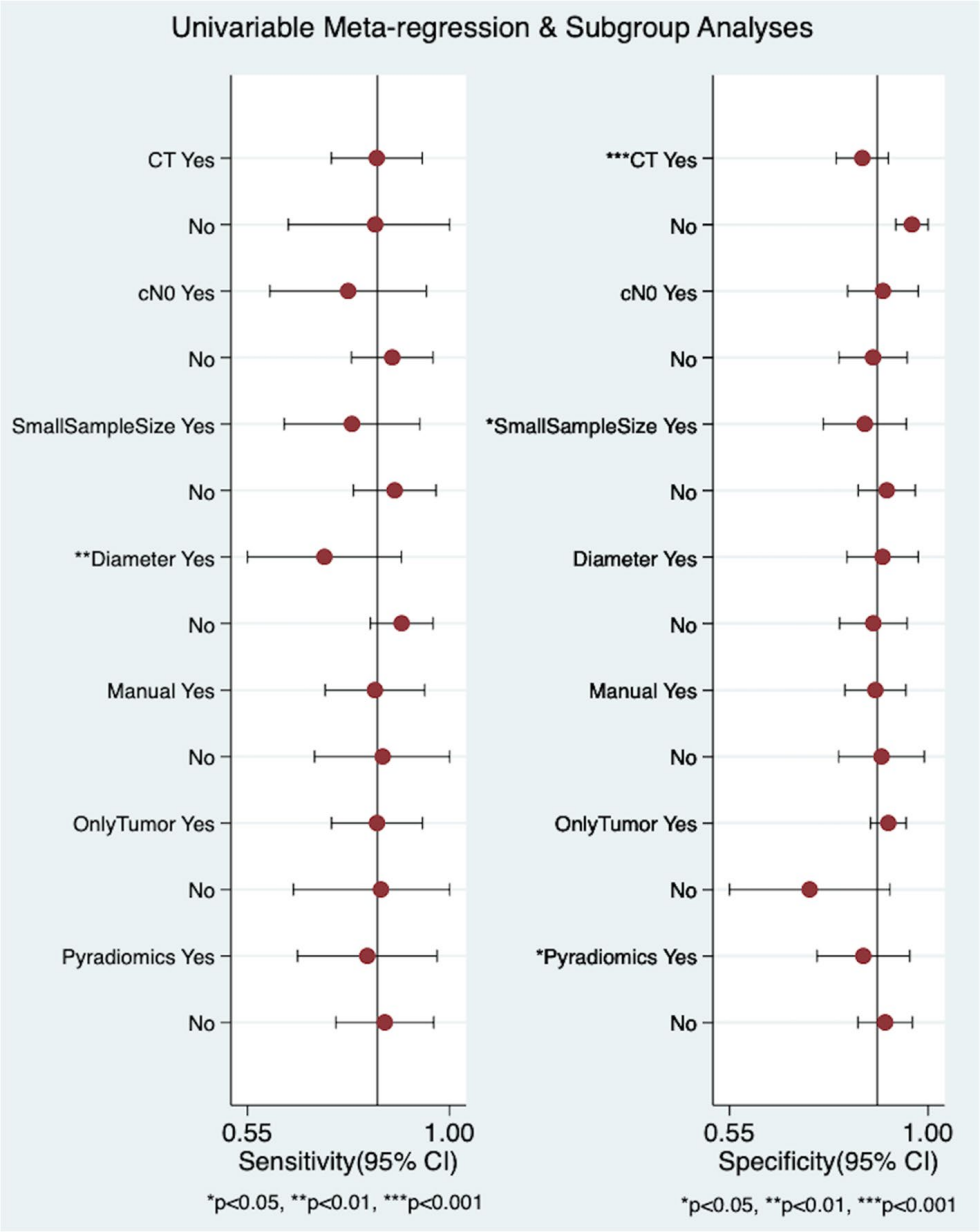
Univariable Meta-regression analysis plot to investigate sources of heterogeneity. (Small Sample Size: sample sizes ≤ 300; Diameter: primary tumor diameter ≤ 30 mm)

Subgroup analysis was performed on the training cohorts of 10 studies, mainly including the following categories: (1) imaging modality: CT, PET/CT; (2) clinical stage: clinical N0, others; (3) sample size: ≤ 300, > 300; (4) primary tumor diameter: ≤ 30 mm, others; (5) segmentation method: manual, semi-automated/automated; (6) ROI: only primary tumor, including peritumoral/lymph node region; (7) radiomic software: Pyradiomics, others. From Table 4, radiomic features based on primary tumor showed high diagnostic performance in predicting LNM of lung adenocarcinoma in all subgroups. Univariable meta-regression analysis further performed, which showed that primary tumor diameter (*p* < 0.01) was a possible source of heterogeneity in sensitivity. Imaging modalities (*p* < 0.001), sample size (*p* < 0.05), and radiomics software (*p* < 0.05) were possible sources of heterogeneity in terms of specificity (Fig. 6).

**Table 4:** Diagnostic performance of subgroup analysis

Subgroup	No. of studies	Sensitivity (95% CI)	Specificity (95% CI)	PLR (95% CI)	NLR (95% CI)	DOR (95% CI)	AUC (95% CI)
**Overall**	10	0.84(0.73–0.91)	0.88(0.81–0.93)	7.0(4.5–11.0)	0.18(0.11–0.31)	39(19–78)	0.93(0.90–0.95)
**Imaging Modality**							
CT	8	0.84(0.70–0.92)	0.87(0.79–0.92)	6.3(4.0–9.8)	0.19(0.10–0.36)	34(15–79)	0.92(0.89–0.94)
PET-CT	2	/	/	/	/	/	/
**Clinical Stage**							
Clinical N0	4	0.78(0.48-0.93)	0.89(0.80-0.95)	7.4(4.1-13.2)	0.24(0.09-0.68)	30(9-102)	0.92(0.89-0.94)
Others	6	0.87(0.79-0.92)	0.87(0.76-0.94)	6.8(3.6-12.8)	0.15(0.10-0.24)	44(20-96)	0.93(0.90-0.95)
**Sample Size**							
≤ 300	5	0.78(0.57–0.91)	0.85(0.75–0.91)	5.3(3.6–7.6)	0.26(0.13–0.52)	21(11–37)	0.89(0.86–0.92)
> 300	5	0.87(0.78–0.93)	0.90(0.80–0.95)	9.0(4.3–18.8)	0.14(0.08–0.25)	65(24–174)	0.95(0.92–0.96)
**Primary Tumor Diameter**							
≤ 30 mm	4	0.73(0.51–0.87)	0.89(0.72–0.96)	6.9(2.8–17.1)	0.31(0.17–0.56)	23(9–56)	0.88(0.85—0.91)
Others	6	0.89(0.80–0.94)	0.87(0.82–0.91)	7.0(4.7–10.5)	0.12( 0.07–0.23)	56(23–136)	0.94 (0.92–0.96)
**Segmentation Method**							
Manual	7	0.84(0.67–0.93)	0.88(0.80–0.93)	6.8(4.1–11.0)	0.19(0.09–0.39)	36(14–94)	0.92(0.90–0.94)
Semi-automated /Autometed	3	/	/	/	/	/	/
**ROI**							
Only primary tumor	8	0.84(0.70–0.92)	0.91(0.85–0.94)	8.9(5.7–13.9)	0.18(0.09–0.34)	51(24–108)	0.94(0.92–0.96)
Including peritumora/LN reigon	2	/	/	/	/	/	/
**Radiomic Software**							
Pyradiomics	4	0.82(0.69–0.90)	0.85(0.74–0.92)	5.4(2.8–10.3)	0.22(0.12–0.39)	25(8–78)	0.90(0.87–0.92)
Others	6	0.86(0.69–0.95)	0.90(0.81–0.95)	8.6(4.8–15.4)	0.15(0.07–0.36)	55(25–122)	0.94(0.92–0.96)

*AUC* area under the curve, *CI* confidence interval, *DOR* diagnostic odds ratio, *NLR* negative likelihood ratio, *PLR* positive likelihood ratio, *ROI* region of interest

## Discussion

This study revealed that radiomic features extracted from the primary tumor have the potential to predict preoperative LNM in lung adenocarcinoma. The QUADAS-2 and RQS tools were applied to assess the risk of bias and the quality of the radiomic method. Meta-analysis was used to quantitatively evaluate the diagnostic performance of the best radiomics models. Obviously, the radiomics models achieved satisfactory diagnostic performance in both the training and validation cohorts. However, the low methodological quality of the systematic review and the high heterogeneity of the quantitative meta-analysis suggest that radiomics models still need to be further improved to better assist the clinical practice.

The clinical diagnosis of positive LNM is usually based on imaging findings (e.g., short axis diameter of lymph nodes > 10 mm on CT, maximum standardized uptake value ≥ 2.5 on PET/CT). However, the subjective factors of manual identification and the limits of the naked eye are highly likely to induce unwanted bias, such as occult LNM [8, 9, 47, 48]. Radiomics can directly extract features from the ROIs of macroscopic images (such as primary tumor, peritumoral area, etc.) for quantitative analysis in a high-throughput manner [49]. In this review, radiomics studies based on the primary tumor were included. Based on the characteristics of the primary tumor, the severity of tumor hypoxia and angiogenic effects of the primary lesion can be identified to evaluate tumor heterogeneity [50]. Cancerous cells within the primary tumor can proliferate by generating new lymphatic vessels in a variety of ways [51] or they can metastasize to the mediastinum through abundant subpleural drainage [37, 52].

The RQS was able to assess the quality of the radiomic methods; however, the best score achieved in the included studies was 16 (44.4%) [23, 40, 41, 43]. The reason for this result was that 17 studies had a low score in each item of the RQS, which meant that there was a lack of standardized workflow for radiomics research (Table 3). In terms of imaging, all studies documented good image protocol quality and multiple segmentations. However, few studies explored the differences between various scanners and provided open data sources, which will lead to low reproducibility of radiomics research. The choice of ROI segmentation method also had a certain effect. The accuracy of manual segmentation is high, but it is limited by time consumption and inter-reader variation. In one study, radiomic features were not included in the best prediction model, likely because only three independent features were selected for analysis due to the small sample size [36]. Skewness was incorporated as a radiomics feature in the best prediction models of 5 studies [30, 34, 35, 38, 43], and one study found that the skewness of lymph node positive lesions was significantly lower than that of negative lesions [30]. Meanwhile, the biological validation of models can facilitate the clinical translation of radiomics. Although two studies combined genes or proteins [44, 45], neither of them was statistically significant. Finally, multi-center validation is an important key to reduce overfitting and optimize the model. Therefore, future radiomics studies would be better follow standardized workflows, such as obtaining large and high-quality multi-center datasets, ensuring consistent image acquisition parameters, developing accurate and reproducible segmentation methods, and correlating with genomics or proteomics.

According to the QUADAS-2 results, most studies were of a low risk and had good applicability, which may be due to the inclusion of appropriate patient groups and the selection of gold standards for reference. However, some studies were unclear about the selection of participants and whether the use of gold standards was made uninformed decisions. Thus, future studies are needed to illustrate the exclusion criteria and procedures for patient selection clearly, as well as whether there is an appropriate time interval between the reference standard and imaging examination.

The high heterogeneity of radiomics models in quantitative evaluation cannot be ignored, although they showed good diagnostic performance. We observed whether the primary tumor was ≤ 30 mm as a possible source of heterogeneity in sensitivity. Tumor diameter was also identified as an important predictor among non-radiomic features in this review (Additional file 1: Table S5) [34, 35, 37, 40, 43]. Similarly, patients with a relatively large primary tumor diameter tend to have a relatively high probability of LNM and poor prognosis [46]. Meanwhile, in terms of specificity, imaging modality, sample size and radiomics software were possible sources of heterogeneity. This review mainly included CT-based radiomics models, and its diagnostic performance compared with other imaging modalities (PET or PET/CT) remains to be studied. One of the included studies compared the performance of radiomic prediction models derived from different imaging modalities (CT, PET, or PET/CT) and showed that PET/CT yielded best results than the other [41]. Larger sample size will allow for a more comprehensive assessment of a radiomics study, and public database could expand the sample size for the study [53]. Different radiomics feature extraction software was used in this review, which led to the heterogeneity in specificity. One study showed that discrepancies were present in seven different radiomics feature extraction software [54]. Therefore, for the differences caused by image acquisition, it is necessary to perform image normalization (such as resampling, etc.) or follow the standardization protocol of image acquisition and reconstruction in further studies [55], which will be of great help to the stability of radiomics feature extraction. In addition, the algorithms and codes of radiomics feature software would be better conform to the image biomarker standardization initiative to improve its reproducibility and verify in multiple cohorts [54].

There were also some limitations in this systematic review. Firstly, almost all the included studies were from China. Therefore, some geographic bias may be present due to the greater prevalence of adenocarcinoma in Asian populations. Secondly, all studies were retrospective, and only three studies used multicenter data. This may lead to selection bias. Third, studies on MRI were not included in this review due to a lack of matching studies. Fourthly, low RQS and high QUADAS-2 results may have some impact on the literature quality assessment. Finally, only 10 of the included articles were used for meta-analysis, and they showed high heterogeneity. Although we found possible sources of heterogeneity, more studies are needed to further explore it in the future.

## Conclusions

In conclusion, this review summarized that radiomic features based on the primary tumor have the potential to predict preoperative LNM of lung adenocarcinoma. However, future research needs standardized radiomics workflow such as multi-center and prospective studies to promote the applicability of radiomics.

### Supplementary Information

**Supplementary Material 1. **

## Data Availability

All data generated or analysed during this study are included in this published article [and its supplementary information files].

## Abbreviations

AUC: Area under the curve
C-index: Concordance index
CI: Confidence interval
DOR: Diagnostic odds ratio
LNM: Lymph node metastasis
NLR: Negative likelihood ratio
PLR: Positive likelihood ratio
PRISMA-DTA: Preferred Reporting Items for Systematic Reviews and Meta-Analyses for Diagnostic Test Accuracy
QUADAS-2: Quality Assessment of Diagnostic Accuracy Studies-2
ROI: Region of interest
RQS: Radiomics quality score
SROC: Summary receiver operating characteristic
