# Supplementary material for: Predictive value of radiomic features extracted from primary lung adenocarcinoma in forecasting thoracic lymph node metastasis: a systematic review and meta-analysis
Source: BMC Pulm Med. 2024 May 18;24:246. doi: 10.1186/s12890-024-03020-x (PMC11102161; doi:10.1186/s12890-024-03020-x)
Supplement: Supplementary file 1 — Supplementary Material 1. [file 12890_2024_3020_MOESM1_ESM.docx]

**Supplementary Material**

**Table S1: Preferred Reporting Items for a Systematic Review and Meta-analysis of Diagnostic Test Accuracy Studies (PRISMA-DTA) Checklist**

| **Section/topic** | **#** | **PRISMA-DTA Checklist Item** |
| --- | --- | --- |
| **TITLE / ABSTRACT** | | |
| Title | 1 | Identify the report as a systematic review (+/- meta-analysis) of diagnostic test accuracy (DTA) studies. |
| Abstract | 2 | Abstract: See PRISMA-DTA for abstracts. |
| **INTRODUCTION** | | |
| Rationale | 3 | Describe the rationale for the review in the context of what is already known. |
| Clinical role of index test | D1 | State the scientific and clinical background, including the intended use and clinical role of the index test, and if applicable, the rationale for minimally acceptable test accuracy (or minimum difference in accuracy for comparative design). |
| Objectives | 4 | Provide an explicit statement of question(s) being addressed in terms of participants, index test(s), and target condition(s). |
| **METHODS** | | |
| Protocol and registration | 5 | Indicate if a review protocol exists, if and where it can be accessed (e.g., Web address), and, if available, provide registration information including registration number. |
| Eligibility criteria | 6 | Specify study characteristics (participants, setting, index test(s), reference standard(s), target condition(s), and study design) and report characteristics (e.g., years considered, language, publication status) used as criteria for eligibility, giving rationale. |
| Information sources | 7 | Describe all information sources (e.g., databases with dates of coverage, contact with study authors to identify additional studies) in the search and date last searched. |
| Search | 8 | Present full search strategies for all electronic databases and other sources searched, including any limits used, such that they could be repeated. |
| Study selection | 9 | State the process for selecting studies (i.e., screening, eligibility, included in systematic review, and, if applicable, included in the meta-analysis). |
| Data collection process | 10 | Describe method of data extraction from reports (e.g., piloted forms, independently, in duplicate) and any processes for obtaining and confirming data from investigators. |
| Definitions for data extraction | 11 | Provide definitions used in data extraction and classifications of target condition(s), index test(s), reference standard(s) and other characteristics (e.g. study design, clinical setting). |
| Risk of bias and applicability | 12 | Describe methods used for assessing risk of bias in individual studies and concerns regarding the applicability to the review question. |
| Diagnostic accuracy measures | 13 | State the principal diagnostic accuracy measure(s) reported (e.g. sensitivity, specificity) and state the unit of assessment (e.g. per-patient, per-lesion). |
| Synthesis of results | 14 | Describe methods of handling data, combining results of studies and describing variability between studies. This could include, but is not limited to: a) handling of multiple definitions of target condition. b) handling of multiple thresholds of test positivity, c) handling multiple index test readers, d) handling of indeterminate test results, e) grouping and comparing tests, f) handling of different reference standards |

| **Section/topic** | **#** | **PRISMA-DTA Checklist Item** |
| --- | --- | --- |
| Meta-analysis | D2 | Report the statistical methods used for meta-analyses, if performed. |
| Additional analyses | 16 | Describe methods of additional analyses (e.g., sensitivity or subgroup analyses, meta-regression), if done, indicating which were pre-specified. |
| **RESULTS** | | |
| Study selection | 17 | Provide numbers of studies screened, assessed for eligibility, included in the review (and included in meta-analysis, if applicable) with reasons for exclusions at each stage, ideally with a flow diagram. |
| Study characteristics | 18 | For each included study provide citations and present key characteristics including: a) participant characteristics (presentation, prior testing), b) clinical setting, c) study design, d) target condition definition, e) index test, f) reference standard, g) sample size, h) funding sources |
| Risk of bias and applicability | 19 | Present evaluation of risk of bias and concerns regarding applicability for each study. |
| Results of individual studies | 20 | For each analysis in each study (e.g. unique combination of index test, reference standard, and positivity threshold) report 2x2 data (TP, FP, FN, TN) with estimates of diagnostic accuracy and confidence intervals, ideally with a forest or receiver operator characteristic (ROC) plot. |
| Synthesis of results | 21 | Describe test accuracy, including variability; if meta-analysis was done, include results and confidence intervals. |
| Additional analysis | 23 | Give results of additional analyses, if done (e.g., sensitivity or subgroup analyses, meta-regression; analysis of index test: failure rates, proportion of inconclusive results, adverse events). |
| **DISCUSSION** | | |
| Summary of evidence | 24 | Summarize the main findings including the strength of evidence. |
| Limitations | 25 | Discuss limitations from included studies (e.g. risk of bias and concerns regarding applicability) and from the review process (e.g. incomplete retrieval of identified research). |
| Conclusions | 26 | Provide a general interpretation of the results in the context of other evidence. Discuss implications for future research and clinical practice (e.g. the intended use and clinical role of the index test). |
| **FUNDING** | | |
| Funding | 27 | For the systematic review, describe the sources of funding and other support and the role of the funders. |

*Adapted From:* McInnes MDF, Moher D, Thombs BD, McGrath TA, Bossuyt PM, The PRISMA-DTA Group (2018). Preferred Reporting Items for a Systematic Review and Meta-analysis of Diagnostic Test Accuracy Studies: The PRISMA-DTA Statement. JAMA. 2018 Jan 23;319(4):388-396. doi: 10.1001/jama.2017.19163.

**Table S2: Search Strategy in PubMed, Embase, Web of science core collection and Cochrane Library**

| **Search Strategy** |
| --- |
|  |
| 1. **PubMed search strategy (n =1168)**   #1 (((((((((((((((((((((((((((((((Lung Neoplasms[MeSH Terms]) OR (Pulmonary Neoplasms[Title/Abstract])) OR (Neoplasms, Lung[Title/Abstract])) OR (Lung Neoplasm[Title/Abstract])) OR (Neoplasm, Lung[Title/Abstract])) OR (Neoplasms, Pulmonary[Title/Abstract])) OR (Neoplasm, Pulmonary[Title/Abstract])) OR (Pulmonary Neoplasm[Title/Abstract])) OR (Lung Cancer[Title/Abstract])) OR (Cancer, Lung[Title/Abstract])) OR (Cancers, Lung[Title/Abstract])) OR (Lung Cancers[Title/Abstract])) OR (Pulmonary Cancer[Title/Abstract])) OR (Cancer, Pulmonary[Title/Abstract])) OR (Cancers, Pulmonary[Title/Abstract])) OR (Pulmonary Cancers[Title/Abstract])) OR (Cancer of the Lung[Title/Abstract])) OR (Cancer of Lung[Title/Abstract])) OR (broncho-pulmonary cancer[Title/Abstract])) OR (bronchopulmonary cancer[Title/Abstract])) OR (Ca lung[Title/Abstract])) OR (carcinogenesis of the lung[Title/Abstract])) OR (lung malignancies[Title/Abstract])) OR (lung malignancy[Title/Abstract])) OR (malignancies of the lung[Title/Abstract])) OR (malignancy of the lung[Title/Abstract])) OR (malignant lung tumor[Title/Abstract])) OR (malignant neoplasm of the lung[Title/Abstract])) OR (malignant tumor of the lung[Title/Abstract])) OR (pulmonary cancer[Title/Abstract])) OR (pulmonary malignancies[Title/Abstract])) OR (pulmonary malignancy[Title/Abstract])  #2 ((((((((((((((((((((((((((((Carcinoma, Non-Small-Cell Lung[MeSH Terms]) OR (Carcinoma, Non Small Cell Lung[Title/Abstract])) OR (Carcinomas, Non-Small-Cell Lung[Title/Abstract])) OR (Lung Carcinoma, Non-Small-Cell[Title/Abstract])) OR (Lung Carcinomas, Non-Small-Cell[Title/Abstract])) OR (Non-Small-Cell Lung Carcinomas[Title/Abstract])) OR (Non-Small Cell Lung Carcinoma[Title/Abstract])) OR (Non-Small-Cell Lung Carcinoma[Title/Abstract])) OR (Non Small Cell Lung Carcinoma[Title/Abstract])) OR (Nonsmall Cell Lung Cancer[Title/Abstract])) OR (Carcinoma, Non-Small Cell Lung[Title/Abstract])) OR (Non-Small Cell Lung Cancer[Title/Abstract])) OR (bronchial non small cell cancer[Title/Abstract])) OR (bronchial non small cell carcinoma[Title/Abstract])) OR (lung cancer, non small cell[Title/Abstract])) OR (lung non small cell cancer[Title/Abstract])) OR (lung non small cell carcinoma[Title/Abstract])) OR (non oat cell lung cancer[Title/Abstract])) OR (non small cell bronchial cancer[Title/Abstract])) OR (non small cell cancer, lung[Title/Abstract])) OR (non small cell lung carcinoma[Title/Abstract])) OR (non small cell pulmonary cancer[Title/Abstract])) OR (non small cell pulmonary carcinoma[Title/Abstract])) OR (non squamous NSCLC[Title/Abstract])) OR (non-oat cell lung cancer[Title/Abstract])) OR (nonsmall cell carcinoma of the lung[Title/Abstract])) OR (nonsmall cell lung cancer[Title/Abstract])) OR (pulmonary non small cell cancer[Title/Abstract])) OR (pulmonary non small cell carcinoma[Title/Abstract])  #3 ((((((((Adenocarcinoma of Lung[MeSH Terms]) OR (Lung Adenocarcinomas[Title/Abstract])) OR (Lung Adenocarcinoma[Title/Abstract])) OR (Adenocarcinoma, Lung[Title/Abstract])) OR (Adenocarcinomas, Lung[Title/Abstract])) OR (adeno-carcinoma of the lung[Title/Abstract])) OR (adenocancer, lung[Title/Abstract])) OR (lung adeno-carcinoma[Title/Abstract])) OR (pulmonary adenocarcinoma[Title/Abstract])  #4 ((((Lung squamous cell carcinoma*[Title/Abstract]) OR (Squamous Cell Carcinoma, Lung[Title/Abstract])) OR (Lung, Squamous Cell Carcinoma[Title/Abstract])) OR (Squamous cell carcinoma of lung[Title/Abstract])) OR (LUSC[Title/Abstract])  #5 ((((((((((((((((Solitary Pulmonary Nodule[MeSH Terms]) OR (pulmonary nodule*[Title/Abstract])) OR (lung nodule*[Title/Abstract])) OR (ground-glass nodule*[Title/Abstract])) OR (solid nodule*[Title/Abstract])) OR (subsolid nodule*[Title/Abstract])) OR (part-solid nodule*[Title/Abstract])) OR (non-solid nodule*[Title/Abstract])) OR (Nodule, Solitary Pulmonary[Title/Abstract])) OR (Solitary Pulmonary Nodules[Title/Abstract])) OR (Nodules, Solitary Pulmonary[Title/Abstract])) OR (Pulmonary Coin Lesion[Title/Abstract])) OR (Lesion, Pulmonary Coin[Title/Abstract])) OR (Lesions, Pulmonary Coin[Title/Abstract])) OR (Coin Lesions, Pulmonary[Title/Abstract])) OR (Pulmonary Coin Lesions[Title/Abstract])) OR (Coin Lesion, Pulmonary[Title/Abstract])  #6 (((((((((Machine Learning[MeSH Terms]) OR (machine learning[Title/Abstract])) OR (radiomic[Title/Abstract])) OR (radiomics[Title/Abstract])) OR (texture[Title/Abstract])) OR (textures[Title/Abstract])) OR (quantitative[Title/Abstract])) OR (computer-aided[Title/Abstract])) OR (deep learning[Title/Abstract])) OR (feature[Title/Abstract])  #7 ((((((((((((((((((((((Lymphatic Metastasis[MeSH Terms]) OR (Lymphatic Metastases[Title/Abstract])) OR (Metastasis, Lymph Node[Title/Abstract])) OR (Lymph node*[Title/Abstract])) OR (lymphatic involvement[Title/Abstract])) OR (lymph-node*[Title/Abstract])) OR (lymphadenopathy[Title/Abstract])) OR (nodal staging[Title/Abstract])) OR (Node, Lymph[Title/Abstract])) OR (Nodes, Lymph[Title/Abstract])) OR (lymph gland metastasis[Title/Abstract])) OR (lymph metastasis[Title/Abstract])) OR (lymphatic node metastasis[Title/Abstract])) OR (lymphnode metastasis[Title/Abstract])) OR (lymphogenic metastasis[Title/Abstract])) OR (lymphogenous metastasis[Title/Abstract])) OR (lymphoid metastasis[Title/Abstract])) OR (metastasis, lymphatic[Title/Abstract])) OR (lymph gland[Title/Abstract])) OR (lymph nodule*[Title/Abstract])) OR (lymphoid nodule*[Title/Abstract])) OR (lymphonodi[Title/Abstract])) OR (lymphonodus[Title/Abstract])  #8 #1 OR #2 OR #3 OR #4 OR #5  #9 #6 AND #7 AND #8   1. **Embase search strategy (n = 2210)**   #1 'lung cancer'/exp 457,491  #2 'pulmonary neoplasms':ti,ab,kw OR 'neoplasms, lung':ti,ab,kw OR 'lung neoplasm':ti,ab,kw OR 'neoplasm, lung':ti,ab,kw OR 'neoplasms, pulmonary':ti,ab,kw OR 'neoplasm, pulmonary':ti,ab,kw OR 'pulmonary neoplasm':ti,ab,kw OR 'lung cancer':ti,ab,kw OR 'cancer, lung':ti,ab,kw OR 'cancers, lung':ti,ab,kw OR 'lung cancers':ti,ab,kw OR 'cancer, pulmonary':ti,ab,kw OR 'cancers, pulmonary':ti,ab,kw OR 'pulmonary cancers':ti,ab,kw OR 'cancer of the lung':ti,ab,kw OR 'cancer of lung':ti,ab,kw OR 'broncho-pulmonary cancer':ti,ab,kw OR 'bronchopulmonary cancer':ti,ab,kw OR 'ca lung':ti,ab,kw OR 'carcinogenesis of the lung':ti,ab,kw OR 'lung malignancies':ti,ab,kw OR 'lung malignancy':ti,ab,kw OR 'malignancies of the lung':ti,ab,kw OR 'malignancy of the lung':ti,ab,kw OR 'malignant lung tumor':ti,ab,kw OR 'malignant neoplasm of the lung':ti,ab,kw OR 'malignant tumor of the lung':ti,ab,kw OR 'pulmonary cancer':ti,ab,kw OR 'pulmonary malignancies':ti,ab,kw OR 'pulmonary malignancy':ti,ab,kw 308,224  #3 'non small cell lung cancer'/exp 198,942  #4 'carcinoma, non-small-cell lung':ti,ab,kw OR 'carcinoma, non small cell lung':ti,ab,kw OR 'carcinomas, non-small-cell lung':ti,ab,kw OR 'lung carcinoma, non-small-cell':ti,ab,kw OR 'lung carcinomas, non-small-cell':ti,ab,kw OR 'non-small-cell lung carcinomas':ti,ab,kw OR 'non-small cell lung carcinoma':ti,ab,kw OR 'non-small-cell lung carcinoma':ti,ab,kw OR 'carcinoma, non-small cell lung':ti,ab,kw OR 'non-small cell lung cancer':ti,ab,kw OR 'bronchial non small cell cancer':ti,ab,kw OR 'bronchial non small cell carcinoma':ti,ab,kw OR 'lung cancer, non small cell':ti,ab,kw OR 'lung non small cell cancer':ti,ab,kw OR 'lung non small cell carcinoma':ti,ab,kw OR 'non oat cell lung cancer':ti,ab,kw OR 'non small cell bronchial cancer':ti,ab,kw OR 'non small cell cancer, lung':ti,ab,kw OR 'non small cell lung carcinoma':ti,ab,kw OR 'non small cell pulmonary cancer':ti,ab,kw OR 'non small cell pulmonary carcinoma':ti,ab,kw OR 'non squamous nsclc':ti,ab,kw OR 'non-oat cell lung cancer':ti,ab,kw OR 'nonsmall cell carcinoma of the lung':ti,ab,kw OR 'nonsmall cell lung cancer':ti,ab,kw OR 'pulmonary non small cell cancer':ti,ab,kw OR 'pulmonary non small cell carcinoma':ti,ab,kw. 125,616  #5 'lung adenocarcinoma'/exp 50,954  #6 'lung adenocarcinomas':ti,ab,kw OR 'adenocarcinoma of lung':ti,ab,kw OR 'adenocarcinoma, lung':ti,ab,kw OR 'adenocarcinomas, lung':ti,ab,kw OR 'adeno-carcinoma of the lung':ti,ab,kw OR 'adenocancer, lung':ti,ab,kw OR 'lung adeno-carcinoma':ti,ab,kw OR 'pulmonary adenocarcinoma':ti,ab,kw OR 'lung squamous cell carcinoma*':ti,ab,kw OR 'squamous cell carcinoma, lung':ti,ab,kw OR 'lung, squamous cell carcinoma':ti,ab,kw OR 'squamous cell carcinoma of lung':ti,ab,kw OR lusc:ti,ab,kw. 12,917  #7 'lung nodule'/exp 26,432  #8 'pulmonary nodule*':ti,ab,kw OR 'lung nodule*':ti,ab,kw OR 'ground-glass nodule*':ti,ab,kw OR 'solid nodule*':ti,ab,kw OR 'subsolid nodule*':ti,ab,kw OR 'part-solid nodule*':ti,ab,kw OR 'non-solid nodule*':ti,ab,kw OR 'nodule, solitary pulmonary':ti,ab,kw OR 'solitary pulmonary nodules':ti,ab,kw OR 'nodules, solitary pulmonary':ti,ab,kw OR 'pulmonary coin lesion':ti,ab,kw OR 'lesion, pulmonary coin':ti,ab,kw OR 'lesions, pulmonary coin':ti,ab,kw OR 'coin lesions, pulmonary':ti,ab,kw OR 'pulmonary coin lesions':ti,ab,kw OR 'coin lesion, pulmonary':ti,ab,kw OR 'solitary pulmonary nodule':ti,ab,kw. 22,812  #9 'machine learning'/exp 346,366  #10 'radiomics'/exp 6,171  #11 'machine learning':ti,ab,kw OR radiomic:ti,ab,kw OR radiomics:ti,ab,kw OR texture:ti,ab,kw OR textures:ti,ab,kw OR quantitative:ti,ab,kw OR 'computer aided':ti,ab,kw OR 'deep learning':ti,ab,kw OR feature:ti,ab,kw 1,389,043  #12 'lymph node metastasis'/exp 167,132  #13 'lymphatic metastases':ti,ab,kw OR 'metastasis, lymph node':ti,ab,kw OR 'lymphatic involvement':ti,ab,kw OR 'lymph node*':ti,ab,kw OR lymphadenopathy:ti,ab,kw OR 'nodal staging':ti,ab,kw OR 'node, lymph':ti,ab,kw OR 'nodes, lymph':ti,ab,kw OR 'lymph gland metastasis':ti,ab,kw OR 'lymph metastasis':ti,ab,kw OR 'lymphatic node metastasis':ti,ab,kw OR 'lymphnode metastasis':ti,ab,kw OR 'lymphogenic metastasis':ti,ab,kw OR 'lymphogenous metastasis':ti,ab,kw OR 'lymphoid metastasis':ti,ab,kw OR 'metastasis, lymphatic':ti,ab,kw OR 'lymph gland':ti,ab,kw OR 'lymph nodule*':ti,ab,kw OR 'lymphoid nodule*':ti,ab,kw OR lymphonodi:ti,ab,kw OR lymphonodus:ti,ab,kw OR 'lymphatic metastasis':ti,ab,kw. 382,588  #14 #1 OR #2 OR #3 OR #4 OR #5 OR #6 OR #7 OR #8 528,542  #15 #9 OR #10 OR #11 1,595,634  #16 #12 OR #13 439,036  #17 #14 AND #15 AND #16 2,210   1. **Web of Science Core Collection search strategy (n = 3649)**   #1 (((((((((((((((((((((((((((((((TS=(Lung Neoplasms)) OR TS=(Pulmonary Neoplasms)) OR TS=(Neoplasms, Lung)) OR TS=(Lung Neoplasm)) OR TS=(Neoplasm, Lung)) OR TS=(Neoplasms, Pulmonary)) OR TS=(Neoplasm, Pulmonary)) OR TS=(Pulmonary Neoplasm)) OR TS=(Lung Cancer)) OR TS=(Cancer, Lung)) OR TS=(Cancers, Lung)) OR TS=(Lung Cancers)) OR TS=(Pulmonary Cancer)) OR TS=(Cancer, Pulmonary)) OR TS=(Cancers, Pulmonary)) OR TS=(Pulmonary Cancers)) OR TS=(Cancer of the Lung)) OR TS=(Cancer of Lung)) OR TS=(broncho-pulmonary cancer)) OR TS=(bronchopulmonary cancer)) OR TS=(Ca lung)) OR TS=(carcinogenesis of the lung)) OR TS=(lung malignancies)) OR TS=(lung malignancy)) OR TS=(malignancies of the lung)) OR TS=(malignancy of the lung)) OR TS=(malignant lung tumor)) OR TS=(malignant neoplasm of the lung)) OR TS=(malignant tumor of the lung)) OR TS=(pulmonary cancer)) OR TS=(pulmonary malignancies)) OR TS=(pulmonary malignancy) 310087  #2 ((((((((((((((((((((((((((((TS=(Carcinoma, Non-Small-Cell Lung)) OR TS=(Carcinoma, Non Small Cell Lung)) OR TS=(Carcinomas, Non-Small-Cell Lung)) OR TS=(Lung Carcinoma, Non-Small-Cell)) OR TS=(Lung Carcinomas, Non-Small-Cell)) OR TS=(Non-Small-Cell Lung Carcinomas)) OR TS=(Non-Small Cell Lung Carcinoma)) OR TS=(Non-Small-Cell Lung Carcinoma)) OR TS=(Non Small Cell Lung Carcinoma)) OR TS=(Nonsmall Cell Lung Cancer)) OR TS=(Carcinoma, Non-Small Cell Lung)) OR TS=(Non-Small Cell Lung Cancer)) OR TS=(bronchial non small cell cancer)) OR TS=(bronchial non small cell carcinoma)) OR TS=(lung cancer, non small cell)) OR TS=(lung non small cell cancer)) OR TS=(lung non small cell carcinoma)) OR TS=(non oat cell lung cancer)) OR TS=(non small cell bronchial cancer)) OR TS=(non small cell cancer, lung)) OR TS=(non small cell lung carcinoma)) OR TS=(non small cell pulmonary cancer)) OR TS=(non small cell pulmonary carcinoma)) OR TS=(non squamous NSCLC)) OR TS=(non-oat cell lung cancer)) OR TS=(nonsmall cell carcinoma of the lung)) OR TS=(nonsmall cell lung cancer)) OR TS=(pulmonary non small cell cancer)) OR TS=(pulmonary non small cell carcinoma) 86041  #3 ((((((((TS=(Adenocarcinoma of Lung)) OR TS=(Lung Adenocarcinomas)) OR TS=(Lung Adenocarcinoma)) OR TS=(Adenocarcinoma, Lung)) OR TS=(Adenocarcinomas, Lung)) OR TS=(adeno-carcinoma of the lung)) OR TS=(adenocancer, lung)) OR TS=(lung adeno-carcinoma)) OR TS=(pulmonary adenocarcinoma) 44260  #4 ((((TS=(Lung squamous cell carcinoma*)) OR TS=(Squamous Cell Carcinoma, Lung)) OR TS=(Lung, Squamous Cell Carcinoma)) OR TS=(Squamous cell carcinoma of lung)) OR TS=(LUSC) 16233  #5 ((((((((((((((((TS=(Solitary Pulmonary Nodule)) OR TS=(pulmonary nodule*)) OR TS=(lung nodule*)) OR TS=(ground-glass nodule*)) OR TS=(solid nodule*)) OR TS=(subsolid nodule*)) OR TS=(part-solid nodule*)) OR TS=(non-solid nodule*)) OR TS=(Nodule, Solitary Pulmonary)) OR TS=(Solitary Pulmonary Nodules)) OR TS=(Nodules, Solitary Pulmonary)) OR TS=(Pulmonary Coin Lesion)) OR TS=(Lesion, Pulmonary Coin)) OR TS=(Lesions, Pulmonary Coin)) OR TS=(Coin Lesions, Pulmonary)) OR TS=(Pulmonary Coin Lesions)) OR TS=(Coin Lesion, Pulmonary) 18025  #6 ((((((((TS=(Machine Learning)) OR TS=(radiomic)) OR TS=(radiomics)) OR TS=(texture)) OR TS=(textures)) OR TS=(quantitative)) OR TS=(computer-aided)) OR TS=(deep learning)) OR TS=(feature) 2370031  #7 ((((((((((((((((((((((TS=(Lymphatic Metastasis)) OR TS=(Lymphatic Metastases)) OR TS=(Metastasis, Lymph Node)) OR TS=(Lymph node*)) OR TS=(lymphatic involvement)) OR TS=(lymph-node*)) OR TS=(lymphadenopathy)) OR TS=(nodal staging)) OR TS=(Node, Lymph)) OR TS=(Nodes, Lymph)) OR TS=(lymph gland metastasis)) OR TS=(lymph metastasis)) OR TS=(lymphatic node metastasis)) OR TS=(lymphnode metastasis)) OR TS=(lymphogenic metastasis)) OR TS=(lymphogenous metastasis)) OR TS=(lymphoid metastasis)) OR TS=(metastasis, lymphatic)) OR TS=(lymph gland)) OR TS=(lymph nodule*)) OR TS=(lymphoid nodule*)) OR TS=(lymphonodi)) OR TS=(lymphonodus) 160679  #8 #1 OR #2 OR #3 OR #4 OR #5 328259  #9 #8 AND #6 AND #7 3649   1. **Cochrane search strategy (n = 60)**   #1 MeSH descriptor: [Lung Neoplasms] this term only 9469  #2 (Cancer, Pulmonary):ti,ab,kw OR (Cancer of Lung):ti,ab,kw OR (Lung Cancer):ti,ab,kw OR (Pulmonary Cancer):ti,ab,kw OR (Cancer of the Lung):ti,ab,kw  29922  #3 (Pulmonary Cancers):ti,ab,kw OR (Lung Cancers):ti,ab,kw OR (Cancers, Pulmonary):ti,ab,kw OR (Cancers, Lung):ti,ab,kw OR (Pulmonary Neoplasm):ti,ab,kw 2640  #4 (Neoplasms, Lung):ti,ab,kw OR (Pulmonary Neoplasms):ti,ab,kw OR (Lung Neoplasm):ti,ab,kw OR (Neoplasm, Lung):ti,ab,kw OR (Neoplasms, Pulmonary):ti,ab,kw 14352  #5 (Neoplasm, Pulmonary):ti,ab,kw OR (broncho-pulmonary cancer):ti,ab,kw OR (bronchopulmonary cancer):ti,ab,kw OR (Ca lung):ti,ab,kw OR (carcinogenesis of the lung):ti,ab,kw 1456  #6 (lung malignancies):ti,ab,kw OR (lung malignancy):ti,ab,kw OR (malignancies of the lung):ti,ab,kw OR (malignancy of the lung):ti,ab,kw OR (malignant lung tumor):ti,ab,kw 2502  #7 (malignant neoplasm of the lung):ti,ab,kw OR (malignant tumor of the lung):ti,ab,kw OR (pulmonary cancer):ti,ab,kw OR (pulmonary malignancies):ti,ab,kw OR (pulmonary malignancy):ti,ab,kw 5885  #8 MeSH descriptor: [Carcinoma, Non-Small-Cell Lung] this term only 5671  #9 (Non-Small-Cell Lung Carcinomas):ti,ab,kw OR (Lung Carcinoma, Non-Small-Cell):ti,ab,kw OR (Carcinoma, Non Small Cell Lung):ti,ab,kw OR (Carcinoma, Non-Small Cell Lung):ti,ab,kw OR (Lung Carcinomas, Non-Small-Cell):ti,ab,kw 7086  #10 (Non-Small Cell Lung Carcinoma):ti,ab,kw OR (Carcinomas, Non-Small-Cell Lung):ti,ab,kw OR (Nonsmall Cell Lung Cancer):ti,ab,kw OR (Non Small Cell Lung Carcinoma):ti,ab,kw OR (Non-Small-Cell Lung Carcinoma):ti,ab,kw 13077  #11 (Non-Small Cell Lung Cancer):ti,ab,kw OR (bronchial non small cell cancer):ti,ab,kw OR (bronchial non small cell carcinoma):ti,ab,kw OR (lung cancer, non small cell):ti,ab,kw OR (lung non small cell cancer):ti,ab,kw 15071  #12 (lung non small cell carcinoma):ti,ab,kw OR (non oat cell lung cancer):ti,ab,kw OR (non small cell bronchial cancer):ti,ab,kw OR (non small cell cancer, lung):ti,ab,kw OR (non small cell lung carcinoma):ti,ab,kw 15558  #13 (non small cell pulmonary cancer):ti,ab,kw OR (non small cell pulmonary carcinoma):ti,ab,kw OR (non squamous NSCLC):ti,ab,kw OR (non-oat cell lung cancer):ti,ab,kw OR (nonsmall cell carcinoma of the lung):ti,ab,kw 5913  #14 (nonsmall cell lung cancer):ti,ab,kw OR (pulmonary non small cell cancer):ti,ab,kw OR (pulmonary non small cell carcinoma):ti,ab,kw 10292  #15 MeSH descriptor: [Adenocarcinoma of Lung] this term only 130  #16 (Adenocarcinomas, Lung):ti,ab,kw OR (Lung Adenocarcinoma):ti,ab,kw OR (Lung Adenocarcinomas):ti,ab,kw OR (Adenocarcinoma, Lung):ti,ab,kw OR (adeno-carcinoma of the lung):ti,ab,kw 2394  #17 (adenocancer, lung):ti,ab,kw OR (lung adeno-carcinoma):ti,ab,kw OR (pulmonary adenocarcinoma):ti,ab,kw 350  #18 (Lung squamous cell carcinoma*):ti,ab,kw OR (Squamous Cell Carcinoma, Lung):ti,ab,kw OR (Lung, Squamous Cell Carcinoma):ti,ab,kw OR (Squamous cell carcinoma of lung):ti,ab,kw OR (LUSC):ti,ab,kw 2232  #19 MeSH descriptor: [Solitary Pulmonary Nodule] this term only 117  #20 (pulmonary nodule*):ti,ab,kw OR (lung nodule*):ti,ab,kw OR (ground-glass nodule*):ti,ab,kw OR (solid nodule*):ti,ab,kw OR (subsolid nodule*):ti,ab,kw 981  #21 (part-solid nodule*):ti,ab,kw OR (non-solid nodule*):ti,ab,kw OR (Nodule, Solitary Pulmonary):ti,ab,kw OR (Solitary Pulmonary Nodules):ti,ab,kw OR (Nodules, Solitary Pulmonary):ti,ab,kw  178  #22 (Pulmonary Coin Lesion):ti,ab,kw OR (Lesion, Pulmonary Coin):ti,ab,kw OR (Lesions, Pulmonary Coin):ti,ab,kw OR (Coin Lesions, Pulmonary):ti,ab,kw OR (Pulmonary Coin Lesions):ti,ab,kw 4  #23 (Coin Lesion, Pulmonary):ti,ab,kw 1  #24 MeSH descriptor: [Machine Learning] this term only 436  #25 (machine learning):ti,ab,kw OR (radiomic):ti,ab,kw OR (radiomics):ti,ab,kw OR (texture):ti,ab,kw OR (textures):ti,ab,kw 4498  #26 (quantitative):ti,ab,kw OR (computer-aided):ti,ab,kw OR (deep learning):ti,ab,kw OR (feature):ti,ab,kw 42979  #27 MeSH descriptor: [Lymphatic Metastasis] this term only 2349  #28 (Lymphatic Metastases):ti,ab,kw OR (Metastasis, Lymph Node):ti,ab,kw OR (Lymph node*):ti,ab,kw OR (lymphatic involvement):ti,ab,kw OR (lymph-node*):ti,ab,kw 13644  #29 (lymphadenopathy):ti,ab,kw OR (nodal staging):ti,ab,kw OR (Node, Lymph):ti,ab,kw OR (Nodes, Lymph):ti,ab,kw OR (lymph gland metastasis):ti,ab,kw  14553  #30 (lymph metastasis):ti,ab,kw OR (lymphatic node metastasis):ti,ab,kw OR (lymphnode metastasis):ti,ab,kw OR (lymphogenic metastasis):ti,ab,kw OR (lymphogenous metastasis):ti,ab,kw 5409  #31 (lymphoid metastasis):ti,ab,kw OR (metastasis, lymphatic):ti,ab,kw OR (lymph gland):ti,ab,kw OR (lymph nodule*):ti,ab,kw OR (lymphoid nodule*):ti,ab,kw 2968  #32 (lymphonodi):ti,ab,kw OR (lymphonodus):ti,ab,kw 3  #33 #1 OR #2 OR #3 OR #4 OR #5 OR #6 OR #7 OR #8 OR #9 OR #10 OR #11 OR #12 OR #13 OR #14 OR #15 OR #16 OR #17 OR #18 OR #19 OR #20 OR #21 OR #22 OR #23 33414  #34 #24 OR #25 OR #26 46449  #35 #27 OR #28 OR #29 OR #30 OR #31 OR #32 15615  #36 #33 AND #34 AND #35 60 |

**Table S3: The radiomics quality score: RQS**

| **Criteria** | | **Points** |
| --- | --- | --- |
| 1 | Image protocol quality - well-documented image protocols (for example, contrast, slice thickness, energy, etc.) and/or usage of public image protocols allow reproducibility/replicability | + 1 (if protocols are well-documented)  + 1 (if public protocol is used) |
| 2 | Multiple segmentations - possible actions are: segmentation by different physicians/algorithms/software, perturbing segmentations by (random) noise, segmentation at different breathing cycles. Analyse feature robustness to segmentation variabilities | +1 |
| 3 | Phantom study on all scanners - detect inter-scanner differences and vendor-dependent features. Analyse feature robustness to these sources of variability | +1 |
| 4 | Imaging at multiple time points - collect images of individuals at additional time points. Analyse feature robustness to temporal variabilities (for example, organ movement, organ expansion/ shrinkage) | +1 |
| 5 | Feature reduction or adjustment for multiple testing - decreases the risk of overfitting. Overfitting is inevitable if the number of features exceeds the number of samples. Consider feature robustness when selecting features | - 3 (if neither measure is implemented)  + 3 (if either measure is implemented) |
| 6 | Multivariable analysis with non radiomics features (for example, EGFR mutation) - is expected to provide a more holistic model. Permits correlating/inferencing between radiomics and non radiomics features | +1 |
| 7 | Detect and discuss biological correlates - demonstration of phenotypic differences (possibly associated with underlying gene–protein expression patterns) deepens understanding of radiomics and biology | +1 |
| 8 | Cut-off analyses - determine risk groups by either the median, a previously published cut-off or report a continuous risk variable. Reduces the risk of reporting overly optimistic results | +1 |
| 9 | Discrimination statistics - report discrimination statistics (for example, C-statistic, ROC curve, AUC) and their statistical significance (for example, p-values, confidence intervals). One can also apply resampling method (for example, bootstrapping, cross-validation) | + 1 (if a discrimination statistic and its statistical significance are reported)  + 1 (if a resampling method technique is also applied) |
| 10 | Calibration statistics - report calibration statistics (for example, Calibration-in-the-large/slope, calibration plots) and their statistical significance (for example, *P*-values, confidence intervals). One can also apply resampling method (for example, bootstrapping, cross-validation) | + 1 (if a calibration statistic and its statistical significance are reported)  + 1 (if a resampling method technique is also applied) |
| 11 | Prospective study registered in a trial database - provides the highest level of evidence supporting the clinical validity and usefulness of the radiomics biomarker | + 7 (for prospective validation of a radiomics signature in an appropriate trial) |
| 12 | Validation - the validation is performed without retraining and without adaptation of the cut-off value, provides crucial information with regard to credible clinical performance | - 5 (if validation is missing)  + 2 (if validation is based on a dataset from the same institute)  + 3 (if validation is based on a dataset from another institute)  + 4 (if validation is based on two datasets from two distinct institutes)  + 4 (if the study validates a previously published signature)  + 5 (if validation is based on three or more datasets from distinct institutes)  *Datasets should be of comparable size and should have at least 10 events per model feature |
| 13 | Comparison to ‘gold standard’ - assess the extent to which the model agrees with/is superior to the current ‘gold standard’ method (for example, TNM-staging for survival prediction). This comparison shows the added value of radiomics | +2 |
| 14 | Potential clinical utility - report on the current and potential application of the model in a clinical setting (for example, decision curve analysis). | +2 |
| 15 | Cost-effectiveness analysis - report on the cost-effectiveness of the clinical application (for example, QALYs generated) | +1 |
| 16 | Open science and data - make code and data publicly available. Open science facilitates knowledge transfer and reproducibility of the study | + 1 (if scans are open source)  + 1 (if region of interest segmentations are open source)  + 1 (if code is open source) + 1 (if radiomics features are calculated on a set of representative ROIs and the calculated features and representative ROIs are open source) |
|  | Total points (36 = 100%) |  |

Lambin P, Leijenaar RTH, Deist TM et al (2017) Radiomics: the bridge between medical imaging and personalized medicine. Nat Rev Clin Oncol 14:749-762

**Table S4: Risk of Bias and Applicability Judgments in Quality Assessment of Diagnostic Accuracy Studies (QUADAS-2)**

| **Domain** | **Patient selection** | **Index test** | **Reference**  **standard** | **Flow and timing** |
| --- | --- | --- | --- | --- |
| **Signalling**  **questions**  **(yes, no, or**  **unclear)** | Was a consecutive or random sample of patients enrolled?  Was a case-control design avoided?  Did the study avoid inappropriate  exclusions? | Were the index test results interpreted  without knowledge of the results of the reference standard?  If a threshold was used, was it prespecified? | Is the the reference standard likely to correctly classify  the target condition?  Were the reference standard results interpreted without  knowledge of the results of the index test? | Was there an appropriate interval between index test  and reference standard?  Did all patients receive a reference standard?  Did all patients receive the same reference standard?  Were all patients included in the analysis? |
| **Signaling questions (yes, no, or unclear)** | Was a consecutive or random sample of patients enrolled?  Was a case–control design avoided?  Did the study avoid inappropriate exclusions? | Were the index test results interpreted without know- ledge of the results of the reference standard?  If a threshold was used, was it prespecified? | Is the reference standard likely to correctly classify the target condition?  Were the reference standard results interpreted without knowledge of the results of the index test? | Was there an appropriate interval between index tests and reference standard?  Did all patients receive a reference standard?  Did all patients receive the same reference standard? Were all patients included in  the analysis? |
| **Risk of bias**  **(high, low,**  **or unclear)** | Could the selection of patients have  introduced bias? | Could the conduct or interpretation of the index test have introduced bias? | Could the reference standard, its conduct, or its interpretation have introduced bias? | Could the patient flow have introduced bias? |
| **Concerns**  **about**  **applicability**  **(high, low,**  **or unclear)** | Are there concerns that the included patients do not match the review question? | Are there concerns that the index test, its conduct, or its interpretation differ from the review question? | Are there concerns that the target condition as defined by the reference standard does not match the review question? | - |

Whiting PF, Rutjes AW, Westwood ME et al (2011) QUADAS-2: a revised tool for the quality assessment of diagnostic accuracy studies. Ann Intern Med 155:529-536

**Table S5: Radiomics features and non-radiomics features included in the best prediction performance.**

| Study ID | Type of radiomics features in the best performance model | Type of non-radiomics features in the best performance model |
| --- | --- | --- |
| 2018 Gu[30] | first order-Skewness; 90th percentile; shape features-Compactness | CEA level |
| 2018 Liu[31] | Pixel Intensity Histogram-Histogram SD Layer 1 | pleural retraction |
| 2018 Yang[32] | first order-10th percentile; robust mean absolute deviation GLCM-Correlation; Energy_2; Idn; Inverse variance; GLRLM-Gray-level NonUniformity; GLSZM-Gray-level non-uniformity normalized_1; Low gray-level zone emphasis Shape-Flatness; least axis; Maximum 2D diameter column; Spherical disproportion; Surface volume ratio | CT-reported LN status |
| 2018 Zhong[33] | First order-Kurtosis; S(0,5)InvDfMom; 135dr_GLevNonU; Area_S(1,1) | / |
| 2019 Wang[34] | GTV_original_shape_sphericity; GTV_gradient_gldm_LargeDependenceLowGrayLevelEmphasis GTV_wavelet.LLL_firstorder_Skewness PTV_wavelet.HLH_glcm_Imc1 PTV_exponential_glszm_ZoneVariance | tumor size, spiculation, pleural retraction, nodule density, CEA, CT-reported LN status |
| 2019 Yang[35] | first order _Energy; Skewness; GLCM_Maximum Probability; GLCM_Joint Entropy; GLSZM_Gray Level Variance | tumor size, CEA level |
| 2020 Zhu[36] | / | AVG |
| 2021 Das[23] | GPTV-3-Correlation_angle90_offset7, glcmentropy_angle135_offset7, Volume CC;  LN-3-Percentile5, GLCMEntropy_AllDirection_offset1_SD, SurfaceVolumeRatio | spiculation, CEA level |
| 2021 Li[37] | primary tumor first- order variance, wavelet transform, gray histogram features, gradient, and lbp.3D.k glszm small-area emphasis; pleura around the tumor-wavelet; square root; logarithm; gradient | tumor size, spiculation |
| 2021 Ran[38] | original_shape_MinorAxisLength;  log-sigma-1-0-mm-3D_glcm_Idn; original_shape_LeastAxisLength;  wavelet-LLL_glcm_SumEntropy;  log-sigma-1-0-mm-3D_gIrlm_LongRunEmphasis;  wavelet-LHH_glcm_Imc1;  log-sigma-4-0-mm-3D_glcm_Correlation;  wavelet-LLH_glrlm_RunLengthNonUniformity | CT-reported LN status, deep learning signature |
| 2021 Wang[39] | first order-Skewness; total energy;  GLDM-small dependence low gray-level emphasis (SDLGLE);  GLRLM-gray level non-uniformity normalized (GLNN) | CEA level, MTV |
| 2021 Zhang[40] | wavelet-HHH. first-order.Kurtosis wavelet-LHH. gldm. DependenceVariance; original. gldm. LargeDependenceEmphasis | tumor size |
| 2022 Chang[41] | CT-Correlation AllDirection offset4 SD; GreyLevelNonuniformity_AllDirection_offset7_SD; MinIntensity; Surface Area; InverseDifferenceMoment_AllDirection _offset4; Percentile90;  PET-Surface VolumeRatio; Range; MeshVolume; ClusterShade_angle135_offset1 | size of the solid component, location of the lesion |
| 2022 Chen[42] | percentile 25^th^; entropy GLCM 10 | pleural thickening, RDW, LMR |
| 2022 Dai[43] | PET-GLRM_SRE; GLZLM_ZP; GLZLM_SZLGE; GLZLM_ZLNU;  CT-HUKurtosis; HUmin; HUQ3; histogram_skewness; GLZLM_GLNU; GLZLM_ZLNU | TMD, LN L/S |
| 2022 Lv[44] | CT-morph_av; stat_median; stat_max; szm_lzlge_3D; ngl_hdhge_2_5D;  PET-cm_energy_3D_avg; szm_szlge_3D; dzm_zdnu_2_5D | age, histological type, C/T ratio, MTV |
| 2022 Ma[45] | log-sigma-4-0mm-3D_glszm_LargeAreaLowGrayLevelEmphasis; original_firstorder_10Percentile; wavelet-LLH_glszm_ZoneVariance; log-sigma-3-0-mm-3D_gldm_DependenceNonUniformity; log-sigma-3-0-mm-3D_glcm_ClusterShade; log-sigma-5-0-mm-3D_glszm_SizeZoneNonUniformity; log-sigma-2-0-mm-3D_glszm_GrayLevelNonUniformity; log-sigma-5-0-mm-3D_ngtdm_Coarseness; log-sigma-5-0-mm-3D_firstorder_Median ; wavelet-LLL_gldm_DependenceVariance; wavelet-LLL_glszm_LargeAreaHighGrayLevelEmphasis; wavelet-HLL_firstorder_90Percentile; wavelet-HLL_gldm_LargeDependenceEmphasis; wavelet-HHL_glszm_LargeAreaEmphasis; wavelet-LHH_glszm_LargeAreaEmphasis; log-sigma-2-0-mm-3D_glszm_ZoneVariance; log-sigma-4-0-mm-3D_glrlm_LongRunEmphasis; original_firstorder_Median | CEA level, consolidation-to-tumor ratio, Air bronchogram, Pleural attachment, CT-reported LN status, Deep learning signature |

*AVG*, average CT values of whole tumor; *CEA*, carcinoembryonic antigen; *GLCM*, gray level co-occurrence matrix; *GLDM*, gray level dependence matrix; *GLNN*, gray level non-uniformity normalized; *GLNU*, gray-level nonuniformity; *GLRLM*, gray level run length matrix; *GLRM_SRE*, gray-level run-length matrix _short-run emphasis; *GLSZM*: gray level size zone matrix; *GLZLM_ZP*, gray-level zone length matrix_zone percentage; *GPTV*, gross and peritumoral volume; *GTV*, gross tumor volume; *LMR*, lymphocyte-to-monocyte ratio; *LN*, lymph node; *L/S*, ratio of long diameter to short diameter; *MTV*, metabolic tumor volume; *NGTDM*, neighbouring gray tone difference matrix; *PTV*, peritumoral volume; *RDW*, red blood cell distribution width; *SDLGLE*, small dependence low gray-level emphasis; *SZLGE*, short-zone low gray-level emphasis; *TMD*, tumor maximum diameter; *ZLNU*, zone length nonuniformity

**Note.** The references in the supplementary table refer to the reference number in the main manuscript.

**Table S6: The Quality Assessment of Studies for Diagnostic Accuracy (QUADAS-2) assessments of included studies.**

| **Study ID** | **Risk of Bias** | | | |  | **Applicability concerns** | | |
| --- | --- | --- | --- | --- | --- | --- | --- | --- |
|  | **Patient Selection** | **Index Test** | **Reference Standard** | **Flow and Timing** |  | **Patient Selection** | **Index Test** | **Reference Standard** |
| 2018 Gu[30] | + | ? | + | ? |  | + | + | + |
| 2018 Liu[31] | + | + | + | + |  | + | + | + |
| 2018 Yang[32] | + | ? | + | + |  | + | + | + |
| 2018 Zhong[33] | + | ? | + | + |  | + | + | + |
| 2019 Wang[34] | + | + | + | + |  | + | + | + |
| 2019 Yang[35] | + | + | + | + |  | + | + | + |
| 2020 Zhu[36] | + | + | + | + |  | + | + | + |
| 2021 Das[23] | + | + | + | + |  | + | + | + |
| 2021 Li[37] | ? | ? | + | ? |  | + | + | + |
| 2021 Ran[38] | + | ? | + | + |  | + | + | + |
| 2021 Wang[39] | + | + | + | + |  | + | + | + |
| 2021 Zhang[40] | + | + | + | + |  | + | + | + |
| 2022 Chang[41] | ? | + | + | + |  | + | + | + |
| 2022 Chen[42] | ? | ? | + | + |  | + | + | + |
| 2022 Dai[43] | + | + | + | + |  | + | + | + |
| 2022 Lv[44] | ? | ? | + | - |  | + | + | ? |
| 2022 Ma[45] | + | + | + | + |  | + | + | + |

**Note.** The risk of bias and applicability concerns of included studies that indicated low, unclear, or high risk are shown in +, ?, and -, respectively. The references in the supplementary table refer to the reference number in the main manuscript.

**Table S7: Diagnostic accuracy test results from studies included in the meta-analysis.**

|  | **Study ID** | **TP** | **FP** | **FN** | **TN** |
| --- | --- | --- | --- | --- | --- |
| Training cohorts | 2018 Yang[32] | 42 | 13 | 7 | 44 |
|  | 2018 Zhong[33] | 74 | 33 | 4 | 381 |
|  | 2019 Wang[34] | 38 | 53 | 11 | 140 |
|  | 2020 Zhu[36] | 15 | 10 | 24 | 207 |
|  | 2021 Das[23] | 35 | 20 | 4 | 54 |
|  | 2021 Zhang[40] | 21 | 16 | 13 | 110 |
|  | 2022 Chang[41] | 75 | 7 | 30 | 259 |
|  | 2022 Chen[42] | 33 | 16 | 2 | 72 |
|  | 2022 Dai[43] | 58 | 26 | 5 | 112 |
|  | 2022 Ma[45] | 121 | 22 | 12 | 334 |
| Validation cohorts | 2018 Yang_internal[32] | 23 | 5 | 2 | 23 |
|  | 2021 Das_internal[23] | 16 | 16 | 1 | 17 |
|  | 2021 Das_external[23] | 29 | 10 | 2 | 12 |
|  | 2021 Zhang_external[40] | 17 | 10 | 4 | 53 |
|  | 2022 Chang_internal[41] | 29 | 2 | 15 | 111 |
|  | 2022 Chen_internal1[42] | 7 | 7 | 0 | 28 |
|  | 2022 Chen_internal2[42] | 8 | 10 | 0 | 24 |
|  | 2022 Dai_internal[43] | 22 | 7 | 5 | 53 |
|  | 2022 Dai_external[43] | 18 | 3 | 0 | 11 |
|  | 2022 Ma_internal[45] | 28 | 1 | 5 | 89 |
|  | 2022 Ma_external[45] | 29 | 4 | 4 | 71 |

*TP*, true positive; *FP*, false positive; *FN*, false negative; *TN*, true negative; *external,* an external validation cohort; *internal*, an internal validation cohort

**Note.** These data were calculated by Review Manager 5.4 through sample size, sensitivity and specificity. The references in the supplementary table refer to the reference number in the main manuscript.

**Table S8: Summary of the area under the receiver operating characteristic curve(AUC) or** **concordance index(C-index) for each study (the training cohorts).**

| **Study ID** | **AUC/**  **C-index** | **95% CI** | **sensitivity** | **speciﬁcity** | **PPV** | **NPV** |
| --- | --- | --- | --- | --- | --- | --- |
| 2018 Yang[32] | 0.871 | 0.804–0.937 | 0.857 | 0.772 | 0.764^*^ | 0.863^*^ |
| 2018 Zhong[33] | 0.972 | / | 0.948 | 0.920 | 0.691^*^ | 0.990^*^ |
| 2019 Wang[34] | 0.862 | 0.793-0.932 | 0.773 | 0.725 | 0.417^*^ | 0.926^*^ |
| 2020 Zhu[36] | 0.724 | / | 0.388 | 0.955 | 0.795 | 0.774 |
| 2021 Das[23] | 0.900 | 0.84-0.96 | 0.900 | 0.730 | 0.638^*^ | 0.933^*^ |
| 2021 Zhang[40] | 0.782 | 0.691–0.874 | 0.618 | 0.873 | 0.568 | 0.894 |
| 2022 Chang[41] | 0.950 | 0.930-0.970 | 0.717 | 0.974 | 0.943 | 0.853 |
| 2022 Chen[42] | 0.929 | / | 0.943 | 0.818 | 0.673^*^ | 0.973^*^ |
| 2022 Dai[43] | 0.924 | 0.887-0.961 | 0.921 | 0.812 | 0.690 | 0.957 |
| 2022 Ma[45] | 0.974 | 0.959-0.988 | 0.910 | 0.938 | 0.846^*^ | 0.965^*^ |

^*^, these data were calculated by Review Manager 5.4 through sample size, sensitivity and specificity.

*AUC*, area under the receiver operating characteristic curve; *C-index*, concordance index; *NPV*, negative predictive value; *PPV*, positive predictive value; *95% CI*, 95% Confidence interval

**Note.** The references in the supplementary table refer to the reference number in the main manuscript.

**Table S9: Summary of the area under the receiver operating characteristic curve (AUC) or concordance index (C-index) for each study (the validation cohorts).**

| **Study ID** | **AUC/**  **C-index** | **95% CI** | **sensitivity** | **speciﬁcity** | **PPV** | **NPV** |
| --- | --- | --- | --- | --- | --- | --- |
| 2018 Yang_internal[32] | 0.856 | 0.745–0.966 | 0.917 | 0.821 | 0.821^*^ | 0.917^*^ |
| 2021 Das_internal[23] | 0.790 | 0.670–0.920 | 0.940 | 0.510 | 0.497^*^ | 0.942^*^ |
| 2021 Das_external[23] | 0.790 | 0.660-0.930 | 0.920 | 0.560 | 0.747^*^ | 0.832^*^ |
| 2021 Zhang_external[40] | 0.813 | 0.787–0.839 | 0.809 | 0.841 | 0.630 | 0.930 |
| 2022 Chang_internal[41] | 0.940 | 0.890–0.970 | 0.656 | 0.978 | 0.955 | 0.805 |
| 2022 Chen_internal1[42] | 0.886 | / | 1.000 | 0.800 | 0.500^*^ | 1.000^*^ |
| 2022 Chen_internal2 [42] | 0.871 | / | 1.000 | 0.706 | 0.445^*^ | 1.000^*^ |
| 2022 Dai_internal[43] | 0.885 | 0.806–0.964 | 0.815 | 0.883 | 0.758^*^ | 0.914^*^ |
| 2022 Dai_external[43] | 0.948 | 0.879–1.018 | 1.000 | 0.786 | 0.857 | 1.000 |
| 2022 Ma_internal[45] | 0.958 | 0.920-0.996 | 0.848 | 0.989 | 0.966^*^ | 0.947^*^ |
| 2022 Ma_external[45] | 0.969 | 0.938-1.000 | 0.879 | 0.947 | 0.880^*^ | 0.947^*^ |

^*^, these data were calculated by Review Manager 5.4 through sample size, sensitivity and specificity.

*AUC*, area under the receiver operating characteristic curve; *C-index*, concordance index; *external,* an external validation cohort; *internal*, an internal validation cohort; *NPV*, negative predictive value; *PPV*, positive predictive value; *95% CI*, 95% Confidence interval

**Note.** The references in the supplementary table refer to the reference number in the main manuscript.
